# Enhancing Medication Adherence in Older Adults: A Systematic Review of Evidence‐Based Strategies

**DOI:** 10.1111/jgs.70257

**Published:** 2025-12-30

**Authors:** Stefano Scotti, Luca Pasina, Carlotta Lunghi, Emanuel Raschi, Andrea Rossi, Elena Olmastroni, Marco Salluzzo, Sara Mucherino, Valentina Orlando, Alessandro Nobili, Enrica Menditto, Elisabetta Poluzzi, Manuela Casula

**Affiliations:** ^1^ Epidemiology and Preventive Pharmacology Service (SEFAP), Department of Pharmacological and Biomolecular Sciences University of Milan Milan Italy; ^2^ IRCCS MultiMedica Sesto San Giovanni Italy; ^3^ Health Policies Research Department, Laboratory of Clinical Pharmacology and Appropriateness of Drug Prescription Istituto di Ricerche Farmacologiche Mario Negri IRCCS Milan Italy; ^4^ Department of Medical and Surgical Sciences University of Bologna Bologna Italy; ^5^ Population Health and Optimal Health Practices Research Unit Centre de Recherche du CHU de Québec‐Université Laval Québec City Canada; ^6^ Department of Life Sciences, Health and Health Professions Link Campus University Rome Italy; ^7^ Center of Pharmacoeconomics and Drug Utilization Research (CIRFF) University of Naples Federico II Naples Italy; ^8^ Department of Pharmacy University of Naples Federico II Naples Italy

**Keywords:** intervention strategies, medication adherence, older adults, systematic review

## Abstract

**Background:**

Medication adherence is essential for achieving favorable health outcomes, particularly in older adults with multiple chronic conditions.

**Objective:**

This systematic review critically appraised current evidence on interventions aimed at enhancing medication adherence in older adults.

**Methods:**

Literature searches were performed in PubMed/MedLine, EMBASE, and Web of Science for articles published up to December 31, 2024. We identified peer‐reviewed studies assessing interventions to improve medication adherence in older adults (≥ 60 years). The primary outcome was intervention effectiveness; secondary outcomes were clinical parameters, disease control, health‐related quality of life, rehospitalization rates, event rates, mortality rates, feasibility, acceptability or satisfaction levels, and overall costs or cost‐effectiveness.

**Results:**

A total of 128 studies was included: 96 randomized controlled trials (RCTs), 16 pre–post studies, 9 non‐RCTs, and 7 longitudinal evaluations. The majority (51.2%) was implemented in primary care. An educational component was present in 56.3% of interventions, a technical component in 47.6%, and an attitudinal component in 32.0%. Only 3.2% of interventions included rewards. Various healthcare professionals, such as pharmacists, nurses, and physicians, were involved in delivering interventions. Most studies reported improved adherence, though some factors, such as high baseline adherence, insufficient intervention intensity, and brief follow‐up limited the effectiveness. Secondary outcomes often included improvements in disease knowledge, patient satisfaction, quality of life, and clinical indicators like blood pressure and HbA1c levels.

**Conclusions:**

Despite most studies showed a positive impact on adherence, a high heterogeneity was highlighted, and effectiveness was mainly observed in the short term.

## Summary

- Key Points
  ○ We conducted a systematic review to describe interventions aimed at improving medication adherence in older adults, and building on the diversity of approaches and settings identified in the literature, we sought to elucidate the strengths and limitations emerging from individual studies.
  ○ Most interventions had a positive impact on adherence, though effectiveness was often limited to short‐ or medium‐term.
  ○ Most studies prioritize individual diseases over adopting a systemic, patient‐centered strategy addressing multiple comorbidities, which represents a clear limitation for patients with multimorbidity and exposed to polypharmacy.
- Why does this paper matter?
  ○ Medication adherence is crucial for managing chronic conditions in older adults. This systematic review provides a comprehensive assessment of existing interventions, identifying key factors that contribute to their success and limitations.

## Graphical abstract

Designing and implementing an intervention aimed at improving adherence in older subjects.

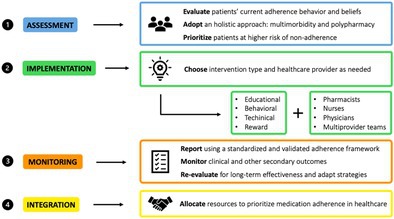

## 1 Introduction

Medication adherence is a critical determinant of health outcomes, particularly among older adults, who often manage multiple chronic conditions with complex medication regimens [1, 2]. In this population, suboptimal adherence to prescribed therapies is well‐documented and contributes substantially to adverse health outcomes [3, 4]. Estimates suggest that up to half of older adults do not take their medications as prescribed, making medication adherence an unresolved and long‐standing challenge [5]. Considering the ongoing global demographic shift, improving treatment adherence is a priority for healthcare systems worldwide [6, 7].

An extensive amount of literature has focused on identifying barriers to medication adherence, and numerous strategies aimed at improving adherence have been developed and tested across various healthcare settings [8]. The first step toward the identification of optimal strategies to promote medication adherence should be based on a review of approaches to date, and a critical evaluation of their strengths and limitations. Previous systematic reviews and meta‐analyses [9, 10, 11] have addressed this topic; however, the pursuit of a quantitative synthesis and the inclusion of only the most robust studies often led to a focus on randomized controlled trials (RCTs), facing considerable difficulty in producing quantitative estimates due to the high degree of heterogeneity in outcome definitions. The development of a framework to guide the selection of the most appropriate intervention, based on patient and setting characteristics, requires broadening the scope of the literature search, embracing the heterogeneity inherent in these strategies and highlighting the multifaceted strengths and weaknesses of each approach. Therefore, we conducted a systematic review to describe interventions intended to enhance adherence to prescribed medications in older adults, on both medication adherence and relevant clinical outcomes, compared with usual care or other active interventions.

## 2 Materials and Methods

### 2.1 Search Strategy

We conducted a systematic review of the literature according to the PRISMA (Preferred Reporting Items for Systematic reviews and Meta‐Analyses) statement guidelines [12] and other available relevant guidelines [13]. The protocol was published in PROSPERO (ID CRD42024575803). PICO framework is illustrated in Table S1. We searched in PubMed/MedLine, EMBASE, and Web of Science for articles published until December 31, 2024. The references of all included articles were also crosschecked. An example of searching strategy is reported in Data S1.

### 2.2 Eligibility Criteria

All peer‐reviewed, original articles reporting any type of intervention aimed at improving medication adherence in older adults were considered for inclusion. In full accordance with the conceptual framework proposed by the ENABLE (European Network to Advance Best practices and technoLogy on medication adherencE) working group, we considered Medication Adherence Enhancing Interventions (MAEIs), defined as “structured activities taking place within, or in association with, the healthcare system that have evidence of a positive effect on medication adherence at the individual patient level” [14].

Eligible study designs included RCTs, non‐randomized controlled trials (NRCTs), pre‐post studies, and observational longitudinal studies. We selected studies where older age (as defined by each study, typically 60 years or older) was among the inclusion criteria, as well as studies where the mean or median patient age was ≥ 60 years. The primary outcome was the change in medication adherence following the intervention, as reported by each study, using its predefined measurement method. Improvement was considered as any significant increase in adherence measure (compared with control groups or baseline). The secondary outcomes were any adherence‐related variables potentially influenced by the intervention, including clinical parameters, disease control, health‐related quality of life (HRQoL), rehospitalization rates, event rates, mortality rates, feasibility, acceptability or satisfaction levels, and overall costs or cost‐effectiveness. We excluded conference proceedings, letters, editorials, commentaries, narrative reviews, consensus statements, and study protocols. Systematic reviews and meta‐analyses were not included as primary studies but were screened for potentially eligible primary studies through their reference lists. Language restriction was applied, including only articles published in English.

### 2.3 Study Selection and Data Processing

The study selection process was conducted by three reviewers (A.R., M.C., and S.S.) independently. Any disagreement among reviewers was resolved through discussion until consensus was reached. All articles were screened for relevance, and those potentially eligible were assessed according to the inclusion/exclusion criteria and accepted or rejected, as appropriate. Titles and abstracts were screened to discard irrelevant papers in the first screening phase. Then, full texts of selected records from the previous step were retrieved and screened to assess their eligibility for inclusion in the qualitative analysis.

For each included article, the following data was extracted: first author, publication year, country, study design (as defined above), sample size, patient age, disease or clinical condition, study setting (primary care: family doctor offices or pharmacies; secondary care: specialist care upon referral; tertiary care: highly specialized care in major hospitals), intervention type (attitudinal: aimed at changing a patient's beliefs, motivations, and thoughts toward adherence; rewards: incentives or recognition to encourage and sustain adherence behaviors; educational; providing information to increase a patient's knowledge about their disease and treatment; technical: practical tools, such as reminders, apps, or pillboxes; see Table S2 for definitions [15, 16, 17]), involved providers (if any: pharmacist, physician, nurse, peer, research staff), follow‐up duration, adherence measurement method, and key findings on primary and secondary outcomes.

### 2.4 Quality Appraisal

The methodological quality and risk of bias of the included studies were assessed using two dedicated tools [18]: the Risk of Bias tool for Interventional Adherence Studies (RoBIAS) for randomized controlled trials (RCTs) and non‐randomized controlled trials (NRCTs), and the Risk of Bias tool for Observational Adherence Studies (RoBOAS) for pre‐post and longitudinal studies. Two reviewers (M.C. and S.S.) independently assessed each domain using a structured ranking scale to determine the risk of bias, categorizing studies as having low, moderate, high, or critical risk of bias [19]. Discrepancies between reviewers were resolved through discussion with a third author. See Data S1 for further details.

## 3 Results

**FIGURE 1:**
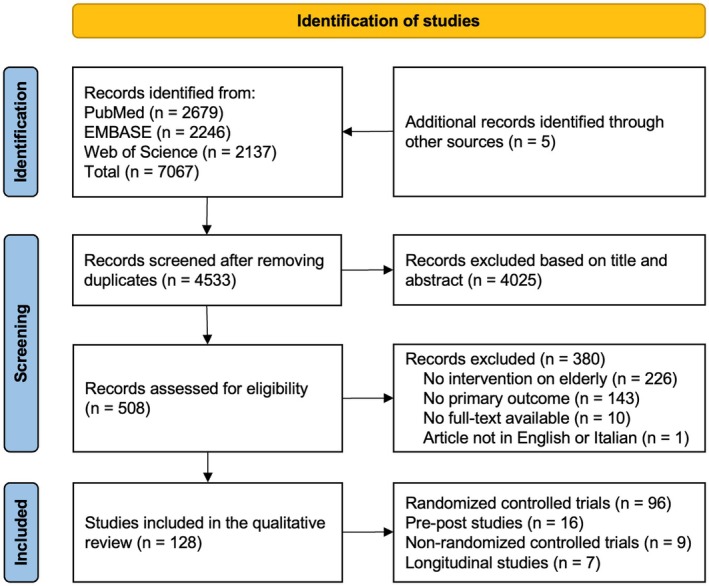
PRISMA diagram of the review's systematic searches.

The full results of the search strategy are depicted in the PRISMA flow diagram (Figure 1). After screening, 128 final studies were identified.

### 3.1 Study Characteristics

We included 96 RCTs (S1–S96 in Supporting Information), 16 pre‐post studies (S97–S112 in Supporting Information), 9 NRCTs (S113–S121 in Supporting Information), and 7 longitudinal evaluations (S122–S128 in Supporting Information), mainly from North America and Europe (Figure S1). Characteristics of included studies are described in Table S3 (for RCTs) and in Table S4 (other studies).

The included studies addressed a wide range of clinical conditions, with the majority (64.3%) focusing on a single chronic disease. Eighteen studies (14.1%) required participants to be on polypharmacy, according to study‐specific definitions. Follow‐up ranged from 10 days to 60 months, with 59.4% of studies presenting a follow‐up of 6 months or less, and 35.2% lasting at least one year.

A single type of intervention was provided in 69.1% of studies. An educational component was present in 56.3% of overall interventions, a technical component in 47.6%, and an attitudinal component in 32.0%. Only 3.2% of interventions included rewards (Figure S2).

Overall, 66 (51.6%) studies were implemented in primary care, 33 (25.8%) in secondary care, and 28 (21.9%) in tertiary care structures. Only one study was conducted in a long‐term care clinic, where a multicomponent intervention (educational + attitudinal + technical) was implemented. The educational component was most commonly applied in secondary and tertiary care settings, while attitudinal interventions and reward‐based approaches were predominantly implemented in primary care (Figure S3).

Almost all the interventions were directed at patients (97.7%), in some cases together with their physicians (3.1%) or their caregivers (3.9%). Two studies evaluated interventions targeted at physicians, while one study focused on pharmacists. Overall, most patient‐directed interventions required the involvement of a healthcare professional: a pharmacist (35.9%), a nurse (18.8%), or a physician (8.6%). In 5.5% of included studies, two healthcare professionals were involved in delivering the intervention. Peer‐delivered interventions were assessed in two studies (1.6%), while trained research staff carried out interventions in 19.5% of cases. In 21.1% of studies, no mediators were required, and interventions were delivered directly to patients (Figure S4).

The attitudinal interventions necessarily required a mediator, and were often delivered through the involvement of nurses, while technical interventions involved a physician or a pharmacist, or were directly delivered to patients (Figure S5). In the primary care setting, the most involved healthcare professional was the pharmacist. Similarly, in secondary care, both physicians and pharmacists collaborated. In the tertiary care setting, nurse mediation was more commonly present (Figure S6).

### 3.2 Results on Primary Outcome

Among 128 studies, 97 (75.8%) reported any improved medication adherence. Notably, studies differed in defined outcomes (continuous adherence or the number of adherent subjects) and, crucially, in the measurement methods, with the majority employing objective methods, such as the proportion of days covered (PDC) (16.4%), pill count (10.9%), or medication possession ratio (MPR) (10.2%). Some studies used automated tools, such as the medication event monitoring system (MEMS) (7.8%). Subjective methods were also employed, including in‐person or telephone questionnaires (15.2%). The 8‐item Morisky medication adherence scale (MMAS‐8) was used in 7.8% of studies, as the 4‐item MMAS (MMAS‐4). Several studies adopted a combined approach.

In the 31 studies (24.2%) reporting no significant effect on adherence, consistent reasons were identified. Hedegaard et al. (S78 in Supporting Information) and Ivers et al. (S16 in Supporting Information) noted that high baseline adherence in the control group limited the intervention's effect. Qvist et al. (S72 in Supporting Information) found a single phone call insufficient for patient engagement, while Tuzun et al. (S44 in Supporting Information) highlighted the lack of evidence on the optimal number of training sessions needed to enhance adherence. Fiscella et al. (S23 in Supporting Information) found no adherence difference between control and intervention groups, attributing this to the large sample size of over 23,000 Medicare patients and nearly 3000 physicians. Kooy et al. (S42 in Supporting Information) emphasized the need for more personalized approaches, as their intervention improved adherence only in women. Tailoring was also supported by Daliri et al. (S69 in Supporting Information), reporting that a nurse‐led multicomponent intervention was effective only in patients not already using a daily multidose drug dispenser. Messerli et al. (S76 in Supporting Information) and Muir et al. (S49 in Supporting Information) pointed out challenges in adherence measurement, as even objective methods could not confirm precisely whether patients took their medications. Finally, some interventions reported some benefits, although no results on medication adherence. Desteghe et al. (S125 in Supporting Information) observed a decline in MMAS‐8 scores and AF‐app usage over three months, despite improved disease knowledge. Oakley et al. (S55 in Supporting Information) found no significant effect on adherence after a four‐month intervention but reported improved treatment decision‐making and patient‐doctor discussions.

### 3.3 Results on Secondary Outcome

Among the 128 included studies, 88 (68.8%) reported at least one secondary outcome, with 50 of these showing positive results. Many studies reported improvements in disease and medication knowledge, satisfaction with the intervention, as well as feasibility and patient acceptability. Cost‐effectiveness evaluations yielded favorable results. Also, improvements in HRQoL, self‐efficacy, disease perception and symptom burden, as well as self‐care, self‐esteem, and attitude were reported. Kamimura et al. (S109 in Supporting Information) highlighted a reduction in caregiver burden following the implementation of a mHealth intervention. Some studies also reported a reduction in medication‐related issues, as well as the number of medications used. Liu et al. (S123 in Supporting Information) observed improvements in diet control and physical activity.

Among clinical indicators, blood pressure control was improved, as well as HbA1c levels, though half of this studies found no improvements in adherence, while lipid level control showed mixed results. Multiple studies also evaluated improvements in depression, anxiety, and neuropsychiatric symptoms or burden. Kolcu et al. (S18 in Supporting Information) reported improvements in body mass index and weight in hypertensive patients, while Aleem et al. (S9 in Supporting Information) described a reduction in intraocular pressure in glaucoma patients.

Fewer studies assessed hard outcomes. Seven studies examined survival and mortality, but with null results. Other studies investigated composite CV events, yet no significant differences were observed between intervention and control groups. Reductions in emergency department visits were reported by Ivers et al. (S16 in Supporting Information) and Murray et al. (S58 in Supporting Information), with only the latter documenting a 19.4% decrease in incidence within the intervention group. Most studies focused on all‐cause rehospitalization or readmission rates, but results were often negligible. Conversely, some studies reported significant reductions in hospitalization rates.

### 3.4 Risk of Bias of Selected Studies

The complete risk of bias assessment for each included study is provided in the Data S1 and in Figures S7–S10. RCTs and NRCTs showed a critical risk of bias in 62.9% of cases, a high risk in 24.8%, a moderate risk in 11.4%, and a low risk in only one study (1.0%). Comparable results were obtained in the assessment of longitudinal and pre‐post studies, with 30.4% of studies classified as having a critical risk of bias, 60.9% at high risk, and 8.7% at moderate risk.

## 4 Discussion

Despite advancements in the last decades [20], medication non‐adherence continues to be an unresolved challenge in clinical practice, particularly among older, multimorbid patients [21, 22]. Building on the diversity of approaches and settings identified in the literature—which mirrors the real‐world context of the research and its field of application—our analysis sought to elucidate the strengths and limitations emerging from individual studies.

### 4.1 Type of Interventions

Patient education is regarded as a main component in enhancing medication adherence. Literature [23] supports the allocation of adequate time for health literacy guidance, coupled with clear and transparent communication regarding the necessity of pharmacological treatments. Although effective, educational interventions address a single layer of the medication adherence issue, and this may limit the effect of the intervention in subjects with a good baseline health literacy (S49 in Supporting Information). Additionally, these strategies are typically associated with an effectiveness that decreases over time, requiring continuous reinforcement (S72 in Supporting Information).

Behavioral and psychosocial factors are another pivotal element of the adherence improvement process, aimed at modifying patients' behavior, habits, and motivation through techniques such as self‐motivation and cognitive restructuring. Available evidence suggests that integrating these strategies within adherence interventions is fundamental to achieve consistent and lasting results [24]. However, these approaches necessarily require specially trained staff and are therefore associated with financial costs, suggesting that their use should be reserved for cases of actual need (S14, S59 in Supporting Information).

The third subgroup includes interventions aimed at facilitating medication intake or increasing the convenience of the medication‐taking process, such as reminders, regimen simplifications, or electronic dispensers. Among them, mHealth approaches, such as mobile or tablet applications, and electronic reminder devices, are recognized as promising tools [25] for improving medication adherence. Recent technological advancements and the availability of affordable, user‐friendly devices have facilitated the implementation of various solutions as tools to enhance adherence. Despite this rising potential, technology‐mediated approaches have occasionally shown inconsistent results (S82, S100, S109 in Supporting Information). Evidence suggests that these systems are effective when poor adherence is due to complex treatment regimens and/or the patient's memory difficulties but are likely ineffective if the issue stems from a lack of belief or trust in the therapy. From this perspective, integrating an educational component could be useful in addressing both aspects concurrently (S27 in Supporting Information).

The fourth type of intervention, which includes incentives or reward systems to promote adherence, was the least common and generally the most challenging to implement and generalize due to financial and organizational burdens.

**FIGURE 2:**
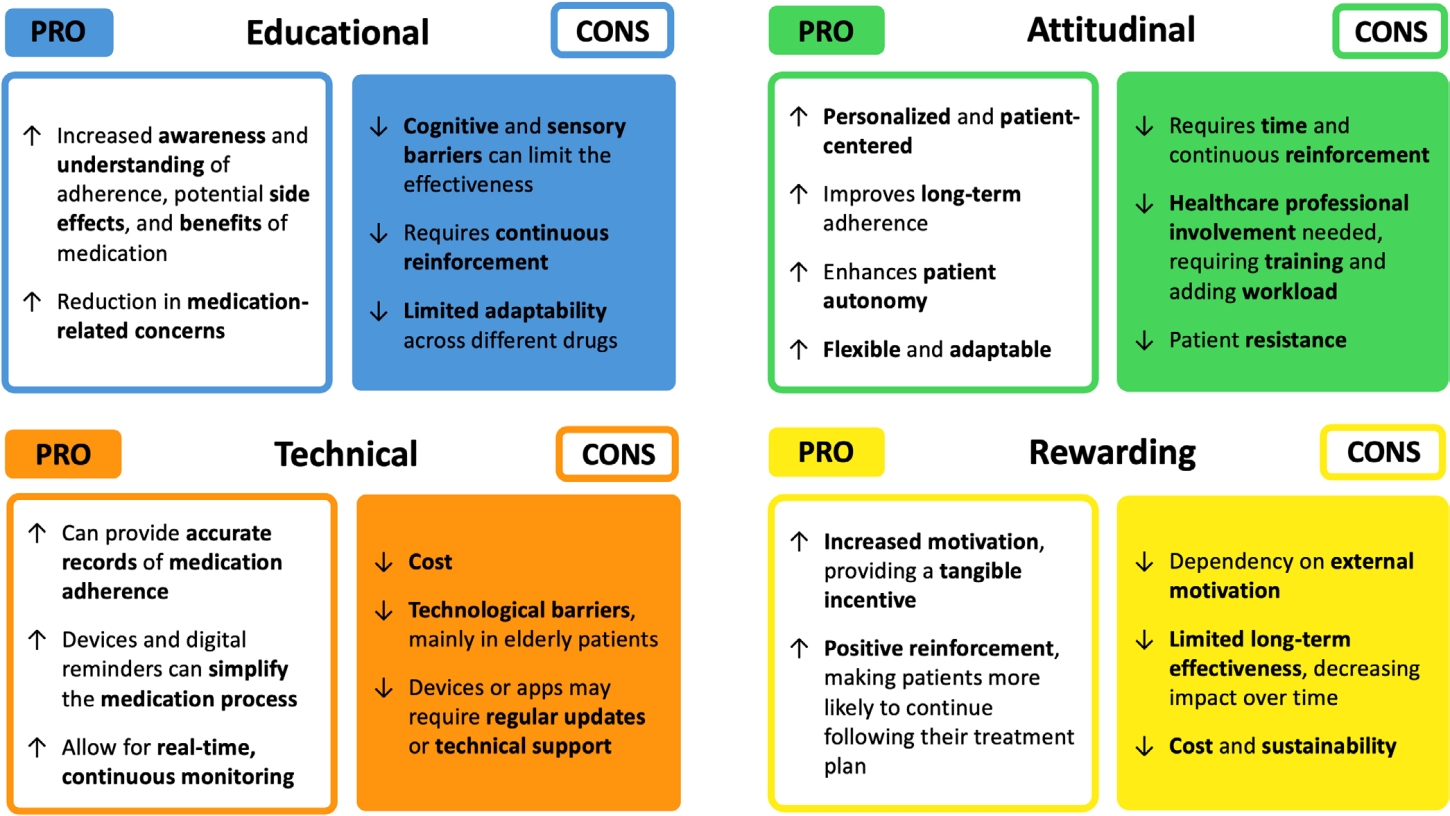
Summary of the main strengths (PRO) and limitations (CONS) of the four components (educational, attitudinal, technical, and rewarding) of adherence‐enhancing interventions in older patients.

Finally, multicomponent interventions combined several adherence‐enhancing strategies simultaneously [26], addressing both patient‐related factors and independent barriers with the potential to achieve synergistic effects. Nevertheless, we observed that multicomponent interventions present drawbacks [27], including excessive sophistication without yielding substantially greater improvements in adherence outcomes [28]. To guide policymakers effectively, it will be crucial to determine whether an optimal balance in multicomponent interventions can be achieved in each setting. Figure 2 visually summarizes the main categories, strengths, and limitations of adherence‐enhancing interventions, as derived from the included studies, developed through a qualitative synthesis and interpretative integration of the evidence.

### 4.2 Providers and Settings

**FIGURE 3:**
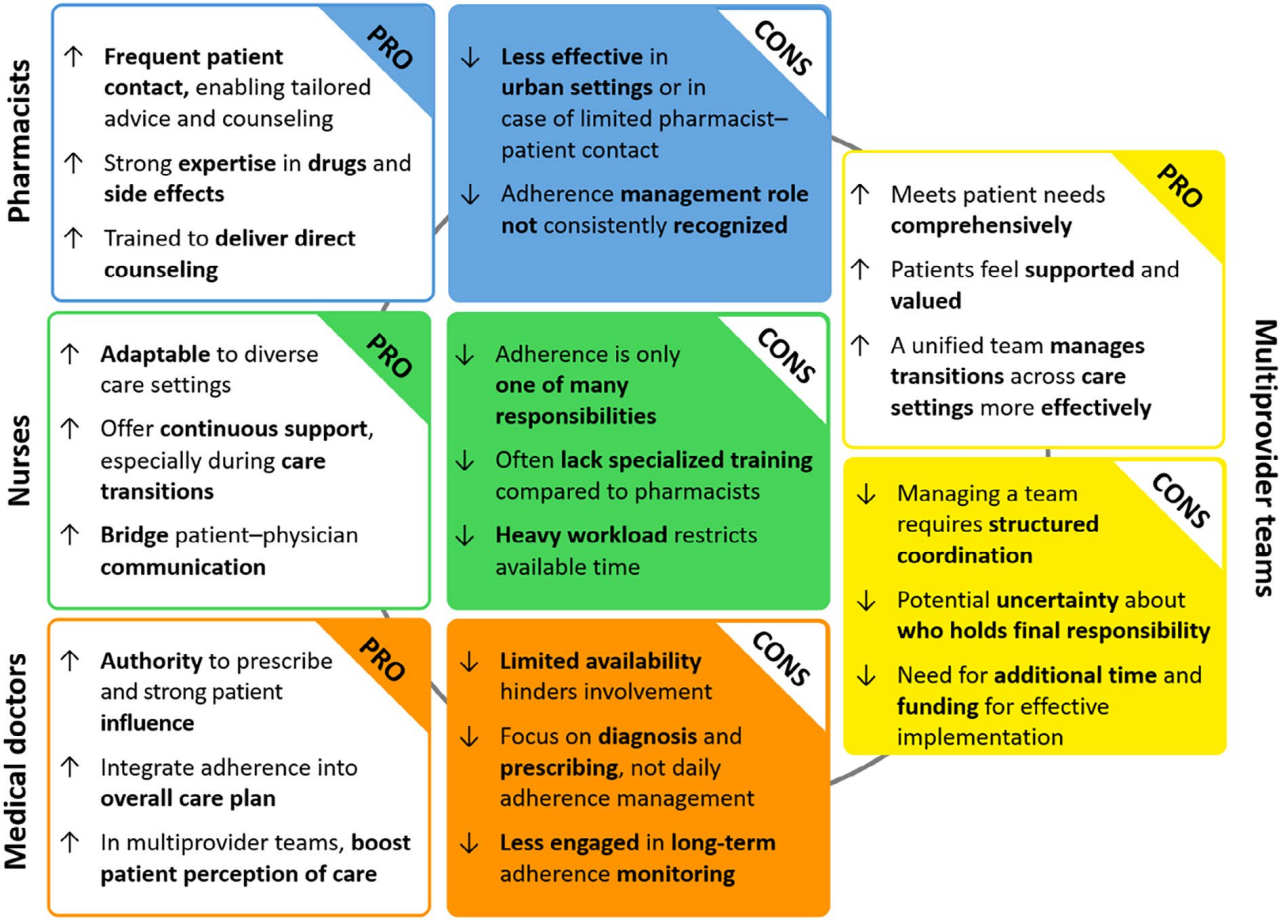
Strengths (PRO) and limitations (CONS) of different healthcare providers in supporting medication adherence.

We highlighted a well‐known ambiguity regarding the responsibility for addressing medication non‐adherence among healthcare professionals. A recent pan‐European survey [29] confirmed that, albeit recognizing the importance of medication adherence, there remains uncertainty over which provider should take the lead when carrying out these strategies. In our review, most interventions were pharmacist‐led, underscoring the value of direct patient engagement with trained providers. However, such approaches may be less feasible in urban areas with limited pharmacist‐patient interaction. Nurses played a key role across diverse settings—hospitals, nursing homes, primary care, specialized services, transitional care, and patients' homes—reflecting their adaptability within healthcare systems and their potential in tackling non‐adherence. Interventions led solely by medical doctors were uncommon, likely due to time and resource constraints that limit their active involvement. Literature suggests that involving multiple providers can enhance patients' perception of care by fostering a sense of support and value, thereby improving adherence when needs are addressed holistically and continuously throughout transitions between healthcare settings [30]. Figure 3 illustrates the complementary strengths and limitations of different providers in supporting medication adherence, based on a qualitative synthesis and interpretative integration of the evidence.

### 4.3 Unaddressed Gaps and Future Directions

In line with previous research, our review found that no single type of adherence‐enhancing intervention consistently outperforms others, as most approaches were associated with at least some improvement in adherence. Although identifying and recommending a universal, one‐size‐fits‐all strategy was beyond the scope of this systematic review, the available literature does not support such a conclusion either. The lack of a clearly superior approach likely reflects the inherent complexity and structural inefficiencies of contemporary healthcare systems, which limit both the design and implementation of adherence interventions. Across the reviewed studies, this fragmentation also hindered the optimization and scalability of human and financial resources.

These findings highlight the need to move toward a more holistic and patient‐centered perspective, shifting away from the traditional, disease‐specific focus that still dominates adherence research. Historically, healthcare professionals and researchers have been constrained by disease‐oriented frameworks [21] that overlook the multifaceted needs of patients with multimorbidity and polypharmacy. Consequently, the complexity of chronic disease management—and the need for integration and coordination across care levels—has often been underestimated.

Future interventions should therefore be tailored to individual needs, engaging patients differently according to their risk of non‐adherence [31, 32]. Achieving this will require new methods and tools to identify at‐risk individuals in a precise and dynamic way. In this regard, emerging data‐driven technologies such as machine learning and artificial intelligence (AI) hold considerable promise [33]. By integrating administrative, clinical, and socioeconomic data, these tools could predict individual adherence trajectories and support personalized action plans. Nevertheless, AI technologies are still at an early stage of development and may generate misleading results or overlook clinically meaningful signals, thereby constraining their current applicability in clinical practice [34].

Beyond AI‐based prediction models, future adherence research should also leverage digital health solutions (e.g., mobile apps, electronic prescribing systems, and wearable sensors) to continuously monitor behavior and provide real‐time feedback. Moreover, co‐designing interventions with patients and healthcare professionals could ensure that solutions are acceptable, feasible, and context‐sensitive. Finally, adopting a multidisciplinary analytical framework—incorporating clinical, behavioral, and organizational factors—may enhance the comparability and external validity of future studies. Taken together, these advances could facilitate the development of adaptable, patient‐centered models of care while improving the efficient allocation of limited healthcare resources.

### 4.4 Strengths and Limitations

To the authors' knowledge, this is the most up‐to‐date and comprehensive evaluation of interventions implemented to improve adherence in the older population. The extensive and in‐depth assessment has highlighted the key strengths and limitations of the different types of interventions.

One of the major limitations of this systematic review was the extreme heterogeneity, which rendered a quantitative meta‐analysis unfeasible. This heterogeneity was evident from the diverse interventions, the different healthcare settings, and the wide range of adherence measurement methods used. Consequently, a statistical measurement of heterogeneity was deemed inappropriate, and we opted for a qualitative synthesis to provide a comprehensive overview of the evidence. Another limitation concerns the overall methodological quality of the included studies. More than two‐thirds were pilot or small‐scale RCTs, often lacking comprehensive reporting on randomization, blinding, or comparators. Indeed, most of the included studies were published before the introduction of dedicated risk‐of‐bias tools for adherence research (RoBIAS and RoBOAS), which established more detailed and stringent assessment criteria. Consequently, when these frameworks were retrospectively applied, many items remained unclear—possibly not because of low study quality per se, but due to incomplete reporting practices that were common at the time. These evident drawbacks should serve as a basis for designing future studies that can ensure more robust methodology and generalizable results. Second, there was substantial inconsistency in the methods used to assess adherence. Although the observed inter‐study variability might not affect the validity of individual studies, the absence of a universally accepted gold standard [35, 36] for measuring non‐adherence is a critical concern when comparing results across studies. In our review, no study explicitly adopted or reported the use of ABC taxonomy [37], thus lacking a carefully selected and validated framework for reporting medication adherence estimates [38]. Third, only a limited number of studies assessed secondary outcomes. Enhancing clinical outcomes and minimizing adverse health effects should be primary objectives alongside the improvement of medication adherence. However, in this systematic review, few studies detected meaningful changes in clinical parameters and disease control, even less in hard outcomes. Addressing these factors is essential for effectively translating adherence research into clinical practice, bridging the current gap between research and real‐world patient care. Finally, much research has overlooked the long‐term persistence of benefits related to adherence. In our review, the majority of the studies had follow‐up periods of ≤ 12 months. A reassessment of long‐term effectiveness, including the potential need for re‐administering the intervention, is often lacking.

## 5 Conclusions

In line with previous research, our systematic review found improvements in medication adherence among older adults across different types of interventions. However, due to the variability observed, it remains challenging to identify clear best practices. Non‐adherence is a complex challenge, and while universal solutions are unlikely, establishing common guidelines or minimum standards is essential. Future efforts should increasingly focus on creating patient‐centered care, with interventions tailored to individual needs. Leveraging technological innovations and standardizing research methodologies will not only enhance our understanding of medication adherence but also help its repositioning as a priority on national healthcare agendas.

## Author Contributions

All authors meet the criteria for authorship stated in the Uniform Requirements for Manuscripts Submitted to Biomedical Journals. E.M., E.P., and M.C. contributed to the concept and design. S.S., A.R., and E.O. were responsible for the acquisition of data. C.L., E.R., M.S., and S.M. contributed to data analysis. L.P., V.O., and A.N. contributed to data interpretation. S.S. and M.C. prepared the draft of the manuscript. All authors contributed to the critical revision of the manuscript.

## Funding

Eldercare (Enabling Medication Adherence in the Elderly) project is a research project aimed at collecting and documenting the most relevant experiences developed in Italy at the national, regional, and local levels to enhance medication adherence in the elderly. The project is funded by the European Union—Next Generation EU (PRIN—Call 2022 Prot. 20227C2YLA).

## Conflicts of Interest

S.S., L.P., C.L., E.R., A.R., E.O., M.S., S.M., V.O., A.N., E.M., E.P., report no disclosures. M.C. received honoraria for lectures from Chiesi, Sobi, and Ultragenyx.

## Supporting information


**Data S1:** Supporting Information.

**Data S2:** Supporting Information.

## Data Availability

The data analyzed during the current study are available from the original published sources cited in the manuscript. No new primary data were generated.
