# Supplementary material for: Enhancing Medication Adherence in Older Adults: A Systematic Review of Evidence‐Based Strategies
Source: J Am Geriatr Soc. 2025 Dec 30;74(2):479–87. doi: 10.1111/jgs.70257 (PMC12911538; doi:10.1111/jgs.70257)

Enhancing medication adherence in older adults: a systematic review of evidence-based strategies

Supplementary Material

Search strategy

The search strategy was developed with headings and keywords used to define the target Population, Intervention, Comparator, and Outcome (PICO) searched as MeSH (Medical Subject Headings) or Emtree terms in the title and abstract. Boolean operators AND/OR were employed.

Search strategy for PubMed

((("adherence"[Title] OR "adhere"[Title] OR "treatment compliance"[Title] OR "pharmacoutilization"[Title] OR "persisten*"[Title]) AND ("patient s"[All Fields] OR "patients"[MeSH Terms] OR "patients"[All Fields] OR "patient"[All Fields] OR "patients s"[All Fields] OR ("patient s"[All Fields] OR "patients"[MeSH Terms] OR "patients"[All Fields] OR "patient"[All Fields] OR "patients s"[All Fields]) OR ("human s"[All Fields] OR "humans"[MeSH Terms] OR "humans"[All Fields] OR "human"[All Fields]) OR ("human s"[All Fields] OR "humans"[MeSH Terms] OR "humans"[All Fields] OR "human"[All Fields]) OR "inpatie*"[All Fields] OR "outpatie*"[All Fields]) AND ("over65"[All Fields] OR "geriat*"[All Fields] OR "olde*"[All Fields] OR "elder*"[All Fields] OR "fragil*"[All Fields] OR ("over"[All Fields] AND "65"[All Fields])) AND (("medicat*"[All Fields] OR "medicin*"[All Fields] OR "therap*"[All Fields] OR "treatmen*"[All Fields] OR "drug"[All Fields] OR ("drug s"[All Fields] OR "pharmaceutical preparations"[MeSH Terms] OR ("pharmaceutical"[All Fields] AND "preparations"[All Fields]) OR "pharmaceutical preparations"[All Fields] OR "drugs"[All Fields]) OR "regime*"[All Fields] OR "remed*"[All Fields]) AND ("intervention*"[All Fields] OR "approac*"[All Fields] OR "education*"[All Fields] OR ("formations"[All Fields] OR "metabolism"[MeSH Terms] OR "metabolism"[All Fields] OR "formation"[All Fields])))) NOT ("case reports"[Publication Type] OR "hascommenton"[All Fields] OR "editorial"[Publication Type] OR "letter"[Publication Type])) AND (english[Filter] OR italian[Filter])

Quality appraisal

RoBIAS and RoBOAS are structured around four domains: (1) study design and implementation, (2) randomization and blinding procedures (RoBIAS) or confounding factors (RoBOAS), (3) adherence outcome measurement and reporting, and (4) data analysis and interpretation. Each domain includes specific items addressing key biases relevant to adherence research, including information bias, reporting bias, selection bias, healthy user bias, insensitive measure bias, confounding bias, and performance bias. Additionally, RoBIAS assesses allocation and ascertainment bias in randomized studies.

For RoBIAS tool, in Domain 1 (study design and implementation of study procedures), substantial gaps were identified. Specifically, 43.2% of studies did not report any data on the consistent delivery of the intervention across study sites and arms, potentially leading to detection bias. Furthermore, baseline adherence assessment, crucial for ensuring an equitable representation of the target population, was entirely absent in 78.3% of studies, indicating a strong selection bias. In Domain 2, performance bias was a major concern. Blinding of participants and researchers to allocation was either unreported or completely absent in 92.5% and 94.3% of studies, respectively. Similarly, researcher blinding during data collection and analysis was critically lacking, with 63.2% and 85.8% of studies, respectively, failing to implement any blinding procedures in these phases. Domain 4 items were often not assessable, particularly those related to methods for handling missing or excluded data (44.3% not applicable), strategies for managing participant loss to follow-up (48.2% not applicable), and inferences on clinical significance (47.2% not applicable).

For RoBOAS tool, in Domain 1, reporting bias stemmed from the lack of a clear description of medication adherence regimens, with this information mostly absent in 26.1% of studies. Additionally, it was not possible to determine whether baseline adherence was assessed during recruitment, as this item was not applicable in 95.7% of studies. Domain 2 contributed to performance bias, particularly due to the lack of adjustment for potential confounders in the analysis. This item was not applicable in 21.7% of studies, completely absent in 8.7%, and mostly absent in 26.1%. Domain 3 highlighted a lack of clear and explicit definitions of adherence phases of interest, with this information mostly absent in 43.5% of studies, possibly contributing to reporting bias. Finally, even in longitudinal and pre-post studies, Domain 4 showed a lack of information on inferences regarding the clinical significance of results, with this item deemed not applicable in 73.9% of studies.

Supplementary Table 1. PICO framework

Data Extraction	Description
Population (P)	Older adults, typically aged 60 or 65 years and above, or adults with a mean/median age of ≥60 years.
Intervention (I)	Any intervention aimed at improving medication adherence, delivered by any provider (e.g., physicians, clinicians, pharmacists, nurses) and implemented in any setting (e.g., nursing homes, hospitals, pharmacies).
Comparator (C)	No intervention or any other intervention and no medication adherence activities.
Outcome (O)	Describing the impact of medication adherence interventions in terms of effectiveness and any other outcomes related to the intervention.

Supplementary Table 2. Category definitions for interventions

Category	Definition
Educational	Interventions providing information regarding the medication, disease state or importance of adherence, in any form (e.g. written, oral, in group, by telephone), to a patient with the aim of increasing a patient's knowledge or skills that facilitate adherence.
Attitudinal	Interventions aiming to modify behavioral intention based on modifying patient's attitudes, beliefs or subjective norm related to their disease state or medication (e.g. motivational interviewing, cognitive behavioral therapy, etc.), delivered in any form (e.g. written, oral, in group, by telephone).
Technical	Interventions providing any gadget, instrument, or system that facilitate the medication intake or increase convenience of the medication taking process, such as reminders, regime simplifications, telephone follow-ups, direction observation therapy, self-monitoring, cue-dose training, electronic monitoring feedback.
Rewards	Interventions that provide incentives, awards or penalties to facilitate medication adherence.

Supplementary Table 3. Characteristics of included randomized controlled trials (RCTs).

Single-disease RCTs										
**First author**	**Year**	**Country**	**Patients (N)**	**Age**	**Disease or condition**	**Setting**	**Intervention**	**Providers**	**Follow-up**	**Adherence measurement tool**
Başoğlu et al.	2024	Turkey	48 (23 I, 25 C)	Mean ± SD 71.2 ± 6.6	Cancer	Tertiary care	Modular booklet lesson, tele-nursing training (videos, messages), WhatsApp reminders (4/week, 4 weeks), and phone monitoring (weeks 5–12).	Nurses	4 months	OCAS
Haider et al.	2024	Canada	47 (24 I, 23 C)	Median (IQR) I: 70 (46-84) C: 69 (51-89)	Multiple myeloma	Secondary care	Structured education (basic knowledge, oral cancer drugs, personalized drug education, material evaluation) vs. written medication information.	Physicians	6 months	MEMS
Ranjbar et al.	2024	Iran	74 (36 I, 38 C)	≥60 as per inclusion criteria	Hypertension	Secondary care	Weekly training (6 weeks), peer-led (intervention) vs. nurse-led (control), Ministry of Health materials, hypertension education, and weekly adherence checklists.	Peers	1.5 months	MMAS-8
Samajdar et al.	2024	India	120	≥60 as per inclusion criteria	T2DM	Primary care	SOC vs. three interventions: smart pillbox (MedpeR®), clinical pharmacological review (CPI), and their combination.	Pharmacists	6 months	Pill count
Wang L et al.	2024	China	445 (223 I, 222 C)	Mean ± SD I: 62.0 ± 13.3 C: 64.3 ± 13.1	HF	Tertiary care	WeChat for medication reminders, education, and follow-ups. The control group received standard care with monthly nurse visits.	Pharmacists	12 months	PDC
Wang Y et al.	2024	China	80 (40 I, 40 C)	Mean ± SD I: 65.4 ± 3.3 C: 65.4 ± 3.5	CHD	Tertiary care	The experimental group received personalized plans, repeated education, and psychological support. The control group received standard care with medication guidance and monthly follow-ups.	Nurses	12 months	Self-reports; Medical staff records
Yoon et al.	2024	South Korea	498 (248 I, 250 C)	Mean ± SD I: 65.7 ± 10.1 C: 65.7 ± 10.5	AF	Tertiary care	The ADHERE-App intervention used a smartphone app for daily medication reminders, BP and HR monitoring, and data tracking. The control group received	Nurses	6 months	Pill count

							standard care and a BP monitor without app access.			
Eimer et al.	2023	Iran	88 (43 I, 45 C)	≥60 as per inclusion criteria	HF	Tertiary care	Pre-discharge training, two-month home follow-up (educational package), phone support (intervention) vs. routine training (control)	None	2 months	Questionnaire
Aleem et al.	2021	Pakistan	100 (50 I, 50 C)	≥60 as per inclusion criteria	Glaucoma	Primary care	Pharmacist-led counseling (every two months for six months), educational materials (brochures, images, medication calendars), eye drop technique demonstrations (videos, live), lifestyle guidance, and WhatsApp adherence reminders.	Pharmacists	6 months	MGLS
Calvo et al.	2021	Spain	119 (54 I, 65 C)	≥75 as per inclusion criteria	MI	Tertiary care	40-minute nursing session (3 months post-hospitalization), medication review, health education on adherence, and reminder call (6 months post-discharge).	Nurses	12 months	Morisky-Green Scale; Hayness-Sacket Scale; Withdrawal of medication from pharmacy
Choi et al.	2021	South Korea	4,962 (1,499 I, 2,279 in group C A and 1,184 in group C B)	≥65 as per inclusion criteria	T2DM	Primary care	Co-payment exemption, appointment reminders, phone calls (missed visits), group education (self-management), and personalized training/counseling (poor HbA1c control).	Research staff	60 months	MPR
Maddison et al.	2021	New Zealand	306 (153 I, 153 C)	Mean ± SD 61.0 ± 11.0	ACS	Tertiary care	Personalized automated SMS (24 weeks), 126 adherence, lifestyle, and stress messages, plus 35 tailored messages (smoking cessation priority).	Nurses	12 months	MPR; MMAS-8
Roshandel et al.	2021	Iran	30 (15 I, 15 C)	Range as per inclusion criteria 60-85	CHD	Primary care	Three 20-minute peer-led educational clips (medication, diet, activities) vs. two 15-minute nurse-led clips (control).	Peers	1 month	Questionnaire
Tzikas et al.	2021	Greece	1,009 (500 I, 509 C)	Median (IQR) I: 75.5 (67-81) C: 76.0 (69-82)	AF	Tertiary care	The intervention included a discharge discussion with patients and family, an informational booklet, access to a	Research staff	12 months	PDC

							helpline, and three follow-up calls at 1 week, 2 months, and 6 months.			
Delavar et al.	2020	Iran	112 (54 I, 58 C)	≥60 as per inclusion criteria	Hypertension	Secondary care	Two 30-45 minute in-person sessions (weeks 1-2), followed by four 15-minute phone sessions. Personalized materials, covering hypertension, medications, adherence, and medical visits. "Teach-back" method used.	Research staff	1 month	MMAS-8
Ivers et al.	2020	Canada	2,632 (878 in each I group, 876 C)	Mean ± SD 66.0 ± 12.4	MI	Tertiary care	The intervention had three groups: usual care (no intervention), a postal group (five mailings), and a combined group (mailings + automated adherence calls).	None	12 months	Self-report; Administrative data
Khosravi et al.	2020	Iran	60 (30 I, 30 C)	≥60 as per inclusion criteria	COPD	Secondary care	Comprehensive care program: in-hospital education, respiratory rehabilitation, home visits, follow-up calls (biweekly for 6 months), monthly home visits, specialist visits (every 3 months), and psychosocial support.	Research staff	6 months	Questionnaire
Kolcu et al.	2020	Turkey	76 (38 I, 38 C)	≥65 as per inclusion criteria	Hypertension	Long-term care	Nurse-led hypertension management program: six education sessions, four weekly motivational meetings, individual/group interventions, action plans, institutional measures (e.g., pillboxes, saltshaker removal), DASH diet, and exercise (active/passive for immobile patients).	Nurses	24 weeks	MMAS-4
Schulz et al.	2019	Germany	237 (110 I, 127 C)	≥60 as per inclusion criteria	CHF	Primary care	Medication review, creation of a therapeutic plan, weekly dosing aid, bi-weekly pharmacy visits for counseling, and monitoring of blood pressure and heart rate. The doctor was contacted for medication issues or significant vital sign changes.	Pharmacists	24 months	PDC
Wu JR et al.	2019	USA	43 patients (23 I, 20 C) and their caregivers	Mean ± SD 66.0 ± 11.0	HF	Tertiary care	45–60-minute in-person session and biweekly follow-up calls for three months, focusing on improving adherence through	Research staff	6 months	MEMS; MMAS-4

							communication, goal setting, and barrier identification.			
Wu Q et al.	2019	China	150 (75 I, 75 C)	≥60 as per inclusion criteria	MI	Tertiary care	A 3-month program with a manual, follow-ups, home visits, and lifestyle/self-management support, including medication guidance. Follow-ups started daily, then reduced to monthly.	Research staff	6 months	Questionnaire
Desteghe et al.	2018	Belgium	48	Mean ± SD 71.6 ± 8.6	AF	Secondary care	Three-phase intervention: 1) daily monitoring with MEMS system, 2) telemonitoring with immediate feedback (phone calls for missed doses or errors), 3) continued monitoring without daily data transmission. Feedback for missed doses or overdoses via phone calls.	None	9 months	Pill count
Fiscella et al.	2018	USA	1,800 patients and 600 physicians	Range as per inclusion criteria 65-89	Glaucoma	Primary care	Educational intervention for patients and physicians via mail.	None	12 months	PDC
Nguyen et al.	2018	Vietnam	166 (79 I, 87 C)	Mean ± SD 61.2 ± 9.6	ACS	Tertiary care	1) 30-minute in-person session (pre-discharge, ACS knowledge, risk factors, medication, personalized guidance, pill organizer), 2) 30-minute phone call (post-discharge, general/medication issues, reinforcement).	Pharmacists	3 months	MMAS-8
Sutema et al.	2018	Indonesia	62 (31 I, 31 C)	≥60 as per inclusion criteria	T2DM	Tertiary care	The intervention group used an app for medication reminders, while the control group received standard therapy without reminders.	None	1 month	Pill count
van der Laan et al.	2018	The Netherlands	170 (85 I, 85 C)	Range 45-75 as per inclusion criteria	Hypertension	Primary care	The intervention group received pharmacist-guided consultations to address adherence barriers and provide tailored recommendations, while the control group received standard care from a pharmacy technician.	Pharmacists	9 months	MARS-5
Guo et al.	2017	China	209 (113 I, 96 C)	Mean ± SD I: 67.4 ± 10.6	AF	Secondary care	mAF app: clinical decision support (risk scores), educational modules (atrial	None	2 months	PQA

				C: 70.9 ± 17.4			fibrillation), self-management tools (heart rate, blood pressure tracking), structured follow-up (reminders at 1, 3, 6, 9, 12 months), and personal health record.			
Lin et al.	2017	Iran	288 (144 I, 144 C)	≥65 as per inclusion criteria	CABG	Tertiary care	Psychoeducation (3 weekly 1-hour sessions with a family member, cardiovascular nurses), motivational interviews (5 weekly 50-minute sessions with trained psychologists), and 4 monthly SMS reminders for medication adherence.	Physicians	18 months	MARS; Pharmacy refill rate
Pandey et al.	2017	Canada	34 (17 I, 17 C)	Mean ± SD<br>I: 64.6 ± 11.5<br>C: 62.1 ± 11.0	MI	Secondary care	Daily SMS reminders at preferred times for the intervention group (medication reminder, no drug details), no SMS for the control group.	None	12 months	Logbooks; PDC
Sirey et al.	2017	USA	231 (115 I, 116 C)	Mean ± SD<br>67.3 ± 8.4	Depression	Primary care	The TIP program included three 30-minute sessions over 6 weeks, focusing on adherence barriers, goal setting, and providing information on depression and antidepressant therapy. Patients were encouraged to consult their doctor.	Research staff	3 months	BMQ
Eyler et al.	2016	USA	30 (16 I, 14 C)	≥65 as per inclusion criteria	Hospitalization	Tertiary care	Motivational interviewing (MI) session led by a pharmacist, alongside standard discharge care: open-ended questions on illness and antibiotics, assessment of adherence barriers, and “Readiness to Change Scale” to evaluate confidence in completing the antibiotic course.	Pharmacists	1 month	Pharmacy contact; Follow-up phone call
Gonnelli et al.	2016	Italy	731 (357 I, 374 C)	Median (IQR)<br>66 (60-73)<br>65 (59-73)	Osteoporosis	Primary care	Group 1 (control): standard medication prescriptions and usual medical explanations. Group 2: standard care plus detailed fracture risk information and a 10-year absolute risk sheet for major osteoporotic fractures.	Physicians	12 months	MMAS-4

Insel et al.	2016	USA	116 (58 I, 58 C)	≥65 as per inclusion criteria	Hypertension	Primary care	Prospective memory strategy: routine establishment, medication-cue association (e.g., breakfast), visualizing intake, using a medication organizer, and taking medication upon remembering. Control group: information on hypertension and fall prevention.	Research staff	6 months	MEMS
Chow et al.	2015	Malaysia	119 (69 I, 50 C)	Mean ± SD 60.3 ± 9.6	T2DM	Primary care	Two home visits by a trained pharmacist: 1) medication use and T2DM management, 2) reinforcement. Intervention group: brochure, food pyramid, medication charts with pictorial labels, and phone reminders for prescription refills. Control group: standard care.	Pharmacists	3 months	MMAS-8
Vinluan et al.	2015	USA	16 (7 I, 9 C)	≥65 as per inclusion criteria	HF	Tertiary care	The control group received standard care with discharge counseling and follow-up calls. The intervention group received personalized pharmacist counseling during hospitalization and follow-up calls at 3, 30, 60, and 90 days, covering HF education and therapy review.	Pharmacists	3 months	MMAS-8
Ganda et al.	2014	Australia	102 (49 group A, 53 group B)	Mean ± SD A: 67.5 ± 11.3 B: 65.9 ± 9.9	Osteoporosis	Secondary care	Group A: continuous management from specialist secondary fracture prevention (SFP) service (baseline, 3, 6, 12, 18, and 24 months). Group B: initial management by SFP service (baseline, 3 months), then care transferred to general practitioner until final visit at 24 months.	Physicians	24 months	MPR
Goldstein et al.	2014	USA	58 (30 for each pillbox group, 28 for each smartphone group)	Mean ± SD 69.0 ± 10.9	HF	Primary care	Four groups: silent smartphone, smartphone with reminders, silent pillbox, pillbox with reminders. Telehealth intervention: electronic pillbox with reminder alarms. m-health intervention: iPhone app with reminders and medication tracking. Control group: smartphones without reminders. All	None	1 month	Pillbox opening; Self-report

							participants received device training and study staff support.			
Goswami et al.	2014	USA	208 (155 I, 53 C)	Mean ± SD<br>I: 69.5 ± 12.3<br>C: 67.8. ± 10.6	Initiating or maintaining statin therapy	Primary care	Integrated program: nursing counseling, adherence information sheet, and optional enrollment in My HeartWise (12-week cholesterol management guide with monthly educational materials). Copay reduction card offered at physician's discretion for commercial insurance.	Nurses	6 months	PDC
Lourenço et al.	2014	Brazil	115 (59 I, 56 C)	Mean ± SD<br>62.0 ± 9.0	CHD	Secondary care	Intervention group: action plans for medication adherence, barrier identification, and strategy development. Two face-to-face meetings and two follow-up phone calls to discuss adherence and challenges.	Research staff	2 months	Percentage of adherence; Global adherence assessment; Dose adequacy; MMAS
O'Connor et al.	2014	USA	2,378 (1,220 I, 1,158 C)	Mean ± SD<br>I: 61.6 ± 13.0<br>C: 62.0 ± 13.3	DM	Primary care	Brief 5-minute phone call from a trained professional to address medication non-adherence, identify and resolve barriers, and provide positive reinforcement for adherence.	Nurses	6 months	MPR
Hadji et al.	2013	Germany	2,740	Mean 65	Breast cancer	Tertiary care	Educational materials (9 letters and brochures) sent by mail during the first year, monthly reminders on endocrine therapy persistence, and gift items. Developed with breast cancer survivors, the intervention was delivered via mail.	None	12 months	Questionnaire
Kooy et al.	2013	The Netherlands	381	≥65 as per inclusion criteria	Initiating statin therapy	Primary care	Counseling group with ERD: written invitation, follow-up phone call (14 days later), 10-minute pharmacist counseling (stages of change model), feedback on dispensing data, discussion on non-adherence, statin benefits, ERD delivery, and follow-up visit details. ERD group:	Pharmacists	12 months	PDC

							received ERD by mail with written instructions. Control group: usual care.			
Rinfret et al.	2013	Canada	300	Mean ± SD 64.0 ± 10.0	Being on antiplatelet therapy after stent implantation	Tertiary care	The intervention involved counseling on antiplatelet therapy post-stent implantation, with follow-up calls at 1 week, 1 month, 6 months, and 9 months to assess adherence and reinforce compliance.	Nurses	12 months	PDC
Tuzun et al.	2013	Turkey	305 (155 I, 150 C)	Mean ± SD 62.4 ± 7.7	Osteoporosis	Primary care	Both groups received a "Starter Training Kit." The intervention group also had four phone calls and four group sessions on osteoporosis, exercise, and nutrition. The control group only received the kit.	Research staff	12 months	Questionnaire
Wong et al.	2013	Hong Kong	274 (113 I, 161 C)	Mean ± SD 62.4 ± 9.4	Hypertension	Primary care	The intervention group received usual care plus a 15–20-minute pharmacist consultation, while the control group only received usual care.	Pharmacists	6 months	MMAS-8
Calvert et al.	2012	USA	108	Median (IQR) 63 (54-71) 62 (52-70)	CHD	Secondary care	Enhanced inpatient counseling, identification of adherence barriers, communication of discharge medications to community pharmacists and primary care physicians, continuous adherence monitoring, and provision of a medication card, pill organizer, and reminder tips.	Pharmacists	6 months	Self-report; PDC
Farmer et al.	2012	UK	211 (126 I, 85 C)	Mean ± SD I: 62.5 ± 11.0 C: 64.1 ± 10.3	T2DM	Secondary care	The intervention combined motivation and action planning: nurse assessed patients' beliefs about adherence (using the Theory of Planned Behavior), reinforced positive attitudes, addressed concerns, and helped patients create specific "if-then" plans for medication intake (where, when, and how).	Nurses	3 months	Electronic medication monitoring device
Kripalani et al.	2012	USA	435	Mean ± SD 63.7 ± 10.4	CHD	Primary care	"Illustrated card" group: visual representation of medication regimen (name, purpose, dosage, color image), updated quarterly and mailed with a	Pharmacists	12 months	CMG

							summary letter, followed by a phone call. "Postcard reminder" group: postcards sent 25 days after last prescription refill to remind patients to renew prescriptions.			
Muir et al.	2012	USA	127 (67 I, 60 C)	Mean ± SD<br>I: 66.0 ± 9.2<br>C: 66.0 ± 10.1	Glaucoma	Tertiary care	Individual session with study coordinator, video on glaucoma, eye drop technique demonstration, and materials tailored to health literacy. Monthly follow-up calls. Control group: standard management and education from ophthalmologist.	Research staff	6 months	DWM
Odegard and Christensen	2012	USA	265 (120 I, 145 C)	Mean ± SD<br>I: 65.0 ± 13.0<br>C: 61.0 ± 13.0	DM	Primary care	Pharmacists called patients overdue for prescription refills, following a standardized script. Calls included assessing medication supply, identifying adherence barriers, providing education and encouragement, and developing a self-management plan. A follow-up call was scheduled within 1 week to 1 month.	Pharmacists	12 months	MPR
Ownby et al.	2012	USA	26	Mean ± SD<br>71.9 ± 5.3	Memory impairment	Tertiary care	Group 1: daily phone calls with a recorded medication reminder, timed according to participant preferences and medication schedule. Group 2: questionnaire to assess information needs, followed by a customized document (based on responses, language, and health literacy).	None	12 months	MEMS
Eussen et al.	2010	The Netherlands	899 (439 I, 460 C)	Mean ± SD<br>I: 60.2 ± 10.9<br>C: 60.1 ± 11.3	Initiating statin therapy	Primary care	Five individual counseling sessions with a pharmacist at key points: first statin prescription, 15 days later, and at 3, 6, and 12 months. Sessions included education on adherence, statins, lipid levels, and an informational letter summarizing key points.	Pharmacists	12 months	Therapy discontinuation
Ruppar et al.	2010	USA	15 (10 I, 5 C)	≥60 as per inclusion criteria	Hypertension	Patients' home	Five components: (1) medication adherence feedback via an electronic pill bottle; (2) blood pressure feedback	Nurses	5 months	MEMS

							linking adherence to control; (3) habit analysis with personalized strategies; (4) medication-taking skills assessment; (5) basic education on medications, dosages, and hypertension risks.			
Sirey et al.	2010	USA	70	≥60 as per inclusion criteria	Depression	Primary care	The TIP program included three 30-minute counseling sessions in the first six weeks, followed by two follow-up calls at 8 and 10 weeks. It focused on assessing adherence barriers, reviewing symptoms and medication, setting goals, and collaborating on a personalized adherence strategy.	Research staff	3 months	Questionnaire
Oakley et al.	2009	UK	33 (16 I, 17 C)	≥65 as per inclusion criteria	Osteoporosis	Primary care	Workshop with a decision aid (booklet, audio, worksheet) and a follow-up consultation with a specialized physician two weeks later, where patients returned completed worksheets.	Research staff	4 months	MARS
Schneider et al.	2008	USA	85 (47 I, 38 C)	≥65 as per inclusion criteria	Hypertension	Secondary care	The intervention provided daily-dose blister packaging (“Pill Calendar”) for the study group, while the control group received medication in traditional pill bottles.	None	12 months	MPR
Smith HD et al.	2008	USA	836 (426 I, 410 C)	Mean ± SD<br>I: 64.7 ± 14.2<br>C: 65.0 ± 13.4	MI	Primary care	Personalized letter, an informational booklet, and a wallet card with medication prompts. It emphasized lifelong β-blocker use and side effect management, with input for primary care physicians.	None	9 months	PDC
Murray et al.	2007	USA	314 (122 I, 192 C)	Mean ± SD<br>I: 61.4 ± 7.7<br>C: 62.6 ± 8.8	HF	Secondary care	Medication history assessment, bi-monthly dispensing with personalized instructions, health literacy-tailored materials, and pharmacist monitoring with data sharing to clinic staff.	Pharmacists	12 months	MEMS
Schroeder et al.	2005	UK	245 (128 I, 117 C)	Mean ± SD<br>I: 67.9 ± 10.3	Hypertension	Primary care	20-minute initial session and a 10-minute follow-up, based on the self-regulation	Nurses	6 months	MEMS

				C: 68.2 ± 9.4			model. Nurses addressed patients' understanding of their diagnosis, treatment concerns, and developed personalized adherence strategies.			
Rosen et al.	2004	USA	33 (16 I, 17 C)	Mean ± SD<br>I: 63.5 ± 9.9<br>C: 62.3 ± 12.3	Receiving metformin prescription	Primary care	Cue-dose training, where patients reviewed electronic cap data with providers, linked medication to routines, and used reminders like alarms. The intervention group received SmartCaps® with time and audible alerts.	None	4 months	MEMS
Bouvy et al.	2003	The Netherlands	152 (74 I, 78 C)	Mean ± SD<br>I: 69.1 ± 10.2<br>C: 70.2 ± 11.2	HF	Secondary care	Structured pharmacy interview, report to the physician, and monthly follow-ups. The control group received usual care.	Pharmacists	6 months	MEMS
Grant et al.	2003	USA	120 (62 I, 58 C)	Mean ± SD<br>I: 64.0 ± 12.0<br>C: 69.0 ± 10.0	T2DM	Secondary care	The intervention involved a pharmacist assessing adherence barriers, providing education, and coordinating with services, with a summary sent to the primary care physician.	Pharmacists	3 months	Questionnaire
Rich et al.	1996	USA	156 (80 I, 76 C)	Mean ± SD<br>I: 80.5 ± 5.7<br>C: 78.4. ± 6.1	HF	Tertiary care	The intervention included education on heart failure, daily nurse meetings, dietary consultations, medication reviews, and post-discharge home visits and nurse follow-ups.	Physicians and nurses	1 month	Pill count
Esposito et al.	1994	USA	41	≥65 as per inclusion criteria	Hospitalizatio n	Secondary care	Four groups: Group 1 received a medication info sheet and discharge instructions; Group 2 received the same, plus 30 minutes of verbal instructions; Group 3 received a written medication schedule with side effects and dosing details; Group 4 received the schedule plus 30 minutes of verbal instructions.	Research staff	2 months	Pill count; Self-reports
**Multimorbidity or polypharmacy RCTs**										
**First author**	**Year**	**Country**	**Patients (N)**	**Age**	**Disease or condition**	**Setting**	**Intervention**	**Providers**	**Time of follow-up**	**Adherence measurement tool**

Solmaz and Altay	2024	Turkey	90 (30 W, 30 T, 30 C)	≥65 as per inclusion criteria	Hypertension with ≥1 antihypertensive drug	Primary care	The W group received hypertension training, a reminder watch, and follow-up consultation. The T group had training and consultation. The C group received only pre- and post-tests.	Research staff	3 months	HCAS; MASES-SF
Jaimalai et al.	2023	Thailand	100 (50 I, 50 C)	≥60 as per inclusion criteria	Hypertension and DM with ≥2 drugs	Primary care	Three-week pharmaceutical literacy program using the Medagogy model, transformative learning, and teach-back strategies to improve medication knowledge, skills, and decision-making.	Research staff	2 months	BMQ
Mohan et al.	2023	USA	720 (240 I, 480 C)	≥65 as per inclusion criteria	DM and hypertension	Primary care	The intervention group received an initial call and five tailored follow-ups from trained pharmacy students. The control group had usual pharmacist care with standard refill reminders.	Pharmacists	12 months	PDC
Poorcheraghi et al.	2023	Iran	184 (92 I, 92 C)	≥60 as per inclusion criteria	Polypharmacy (≥5 drugs)	Secondary care	The intervention used an Android-compatible medication management app with a user-friendly interface, adjustable font, color coding, audio playback, images, and personalized reminders.	None	2 months	MMAS-8
Daliri et al.	2022	The Netherlands	198 (99 I, 99 C)	≥70 as per inclusion criteria	Frailty or hospitalization in the previous 6 months	Tertiary care	Community nurses identified MRPs during home visits, with pharmacists providing recommendations. Visits occurred at 2 days, 1 week, 3 weeks, and 6 weeks post-discharge, using the "Adapted Red Flag" tool for MRPs and non-adherence.	Pharmacists and nurses	1.5 months	PDC
Yang et al.	2022	China	88 (48 I, 40 C)	≥60 as per inclusion criteria	Multimorbidity (≥3 chronic conditions)	Primary care	The six-week program included three in-person sessions (30–40 min) and two weekly follow-up calls (15 min). Sessions covered self-management gaps, MI for beliefs, and adherence strategies.	Nurses	3 months	MARS
Dong et al.	2021	USA	129,820 (32,455 I, 97,365 C)	≥65 as per inclusion criteria	Alzheimer's disease, DM, hypertension,	Primary care	The intervention was an annual pharmacist consultation for the beneficiary or caregiver, with a standardized summary.	Pharmacists	6 months	PDC

					hyperlipidemia					
Qvist et al.	2020	Denmark	1,446 (725 I, 721 C)	Mean ± SD I: 69.1 ± 2.9 C: 69.0 ± 2.8	Abdominal aortic aneurysm (AAA), peripheral arterial disease (PAD) or hypertension	Primary care	The intervention was a semi-structured phone consultation by a study nurse three months post-screening, covering prescriptions, renewals, issues, and advice, with follow-up for non-adherence.	Nurses	60 months	PDC
Raj et al.	2020	India	50 (25 I, 25 C)	≥60 as per inclusion criteria	Non-communicable diseases (DM, hypertension, CHD)	Tertiary care	The intervention included education, a medication diary, and phone reminders. Adherence barriers were identified and addressed through personalized strategies over six months.	Research staff	6 months	Pill count
Zárate-Bravo et al.	2020	Mexico	16 (8 I, 8 C)	≥60 as per inclusion criteria	Polypharmacy (≥3 drugs) and cognitive impairment	Primary care	The intervention used a tablet-based system with abstract reminders, interactive animations, auditory/visual cues, and NFC tracking. It was personalized to prescriptions and introduced by research assistants.	Nurses	3 months	Pill count; DosageMAD; MAQ-8 questionnaire
Shim et al.	2018	Malaysia	152 (73 I, 79 C)	≥65 as per inclusion criteria	Polypharmacy (≥5 drugs)	Tertiary care	Pharmaceutical care program, where a pharmacist collaborated with physicians to review medications, provide counseling, address non-adherence, and discuss pharmaceutical issues.	Physicians and pharmacists	6 months	MALMAS
Messerli et al.	2016	Switzerland	450 (218 I, 232 C)	Mean ± SD I: 67.2 ± 11.5 C: 67.1 ± 11.6	Polypharmacy (≥4 drugs)	Primary care	The intervention group received a PMC at T-0 and T-28, with documentation of recommendations. The control group had a PMC only at T-28, with no documented consultations.	Pharmacists	6 months	MPR; DPPR
Moral et al.	2015	Spain	154 (70 I, 84 C)	≥65 as per inclusion criteria	Polypharmacy (≥5 drugs),	Primary care	The intervention group received 20 hours of MI training to enhance motivation and address non-adherence. The control	Physicians and nurses	6 months	% of drugs consumed vs. prescribed

					≥1 chronic disease		group used an informational approach with persuasive strategies and advice.			
Hedegaard et al.	2014	Denmark	516 (231 I, 285 C)	Median (IQR) 62 (54-68) 60 (52-68)	Ischemic stroke or TIA	Secondary care	Targeted medication review, an MI-based discussion, and three follow-up calls within six months post-discharge. The 30-minute in-person interview was followed by a written summary of the objectives.	Pharmacists	12 months	MPR
Mira et al.	2014	Spain	99 (51 I, 48 C)	≥65 as per inclusion criteria	Multimorbidity and polypharmacy	Primary care	The experimental group used the app with training, while the control group received standard information. The app provided reminders, tracked adherence, and allowed caregiver communication.	None	3 months	MMAS-4
Vollmer et al.	2014	USA	21,752	Mean ± SD UC: 63.6 ± 12.2 IVR: 63.6 ± 12.1 IVR+: 63.5 ± 12.2	T2DM or ASCVD	Primary care	Two arms: IVR, with automated calls for reminders and renewals, and IVR+, which added personalized letters, live calls, EMR feedback, and additional materials. Providers included the IVR system, pharmacy staff, and EMR system.	None	12 months	PDC
Wald et al.	2014	UK	301 (150 I, 151 C)	Median (IQR) 60 (54-68) 61 (49-69)	Hypertension and /or hypercholesterolemia	Primary care	The "Text" group received personalized messages on medication schedules, with daily messages for 2 weeks, then every other day, and weekly for 22 weeks, requiring responses on medication intake.	None	6 months	Interviews
Brath et al.	2013	Austria	53	Mean ± SD 69.4 ± 4.8	T2DM, hypercholesterolemia, or hypertension	Primary care	The intervention used an mHealth-based system with electronic blister packs, NFC phones, and SMS reminders to track and improve medication adherence. Patients with <70% adherence were contacted by phone.	None	13 months	mAMS
Ho et al.	2013	USA	241 (122 I, 119 C)	Mean ± SD I: 63.8 ± 9.3 C: 64.0 ± 8.6	MI or unstable angina	Secondary care	The intervention included medication reconciliation, patient education, pharmacist-doctor collaboration, and	Physicians and pharmacists	12 months	PDC

							reminder calls for medication and renewals.			
Olesen et al.	2013	Denmark	517 (253 I, 264 C)	≥65 as per inclusion criteria	Polypharmacy (≥5 drugs)	Primary care	Initial home visit by a pharmacist to review medications and provide information, followed by three follow-up calls at 3, 6, and 9 months to address issues and medication changes.	Pharmacists	12 months	Pill count
Williams et al.	2012	Australia	75 (36 I, 39 C)	Mean ± SD<br>I: 68.0 ± 8.3<br>C: 66.0 ± 10.8	DM, CKD and hypertension	Secondary care	The intervention approach included daily blood pressure monitoring, an individualized medication chart, a 20-minute DVD on adherence, biweekly motivational calls, and a 1-10 scale to assess confidence in medication-taking.	Nurses	9 months	Pill-count; MMAS-4
Obreli-Neto et al.	2011	Brazil	194 (97 I, 97 C)	≥60 as per inclusion criteria	DM and/or hypertension	Primary care	The intervention included biannual pharmacist follow-ups, group education on adherence, and personalized care plans addressing non-adherence, medication roles, and reminders.	Pharmacists	36 months	Morisky-Green Scale; Computerized history
Elliott et al.	2007	UK	205 (87 I, 118 C)	Median (IQR)<br>67 (34-85)<br>67 (28-88)	Stroke, CVD, asthma, T2DM or RA	Primary care	The intervention involved a pharmacist call two weeks after starting new medication, using an SRM-based interview to discuss adherence and provide guidance.	Pharmacists	2 months	Self-report
Lee JK, et al.	2006	USA	159 (83 I, 76 C)	≥65 as per inclusion criteria	Polypharmacy (≥4 daily drugs)	Tertiary care	The intervention included individualized education with standardized cards, medications in blister packs, and follow-up with clinical pharmacists every 2 months.	Research staff	14 months	Proportion of medications taken
Krass et al.	2005	Australia	182 (100 I, 82 C)	Mean ± SD<br>I: 64.0 ± 9.0<br>C: 65.0 ± 10.0	T2DM with polypharmacy (≥3 drugs)	Primary care	The intervention included monthly pharmacist contacts, adherence assessments, medication reviews, feedback, education, and regular follow-ups.	Pharmacists	9 months	BMQ
Sturgess et al.	2003	UK	110 (75 I, 35 C)	≥65 as per inclusion criteria	Polypharmacy (≥4 drugs)	Primary care	The intervention involved pharmacists assessing medication issues, creating personalized plans with education and	Pharmacists	18 months	Questionnaire; Refill adherence

							adherence strategies, and conducting home visits. The control group received standard services.			
Volume et al.	2001	Canada	292	≥65 as per inclusion criteria	Polypharmacy (≥3 drugs)	Primary care	The intervention included comprehensive care with interviews, follow-ups, and the tool for the treatment group, while the control group received standard care.	Pharmacists	13 months	Questionnaire
Faulkner et al.	2000	USA	30 (15 I, 15 C)	Mean ± SD<br>I: 64.0 ± 12.0<br>C: 61.0 ± 12.0	CABG or PTCA	Tertiary care	The intervention involved weekly phone calls from a pharmacist for 12 weeks to reinforce therapy, verify refills, discuss payment, assess side effects, and identify non-adherence reasons.	Pharmacists	24 months	Pill count; Pharmacy refill records
Solomon et al.	1998	USA	133 with hypertension (63 I, 70 C) and 98 with COPD (43 I, 55 I)	Hypertension<br>Mean ± SD<br>I: 66.3 ± 10.0<br>C: 67.3 ± 11.0<br>BPCO<br>Mean ± SD<br>I: 69.3 ± 5.9<br>C: 69.3 ± 9.2	Hypertension and COPD	Secondary care	The intervention involved patient-centered care with pharmacist-physician collaboration, focusing on assessment, education, and adherence. The control group received standard care.	Pharmacists	6 months	Questionnaire; Pill count
Lowe et al.	1995	UK	79 (42 I, 37 C)	Mean (Range)<br>77 (57-96)<br>79 (59-93)	Multimorbidity and polypharmacy (≥1 drugs)	Secondary care	The intervention had a self-administration group progressing to self-management, while the control group received medications from nursing staff with discharge reminders.	Pharmacists and nurses	10 days	Pill count
Lipton and Bird	1994	USA	706 (350 I, 356 C)	≥65 as per inclusion criteria	Hospitalization and polypharmacy (≥3 drugs)	Secondary care	Pharmacists reviewed medical records, consulted with patients and physicians, provided booklets, and held face-to-face consultations before discharge to discuss medications and issues.	Pharmacists	3 months	Telephonic interview
Lourens and Woodward	1992	Australia	97 (49 A, 48 B)	Mean ± SD<br>A: 71.1 ± 6.1<br>B: 71.3 ± 8.6	Polypharmacy (mean ≥6 daily drugs)	Secondary care	Group A received counseling from a pharmacist with instructions and adherence encouragement. Group B received the same counseling, plus a medication card with detailed drug information.	Pharmacists	1 month	Pill count

AAA = Abdominal aortic aneurysm; ACS =Acute coronary syndrome; AF = Atrial fibrillation; BMQ = Brief medication questionnaire; BP = Blood pressure; CABG = Coronary artery bypass grafting; CHD = Coronary heart disease; CMG = Cumulative medication gap; COPD = Chronic obstructive pulmonary disease; CVD = Cardiovascular disease; DASH = Dietary approaches to stop hypertension; DM = Diabetes mellitus; DVD = Digital video disc; DWM = Days without medication; EMR = Electronic medical record; ERD = Electronic reminder device; GBTM = Group-based trajectory modeling; GP = General practitioner; HbA1c = Hemoglobin type 1c; HCAS = Healthcare adherence scale; HF = Heart failure; HR = Heart rate; IVR = interactive voice recognition; MAD = Medication ambient display; MALMAS =Malaysian medication adherence scale; mAMS = Medication adherence management system; MARS = Medication adherence rating scale; MASES-SF = Morisky adherence self-efficacy scale – short form; MAQ = Medication adherence display; MEMS = Medication event monitoring system; MGLS = Morisky-Green-Levine scale; MI = Myocardial infarction; MMAS-4 = Four-item Morisky medication adherence scale; MMAS-8 = Eight-item Morisky medication adherence scale; MPR =Medication possession ratio; MR = Medication review; MRP = Medication related problems; NFC = near-field communication; OCAS = Oral chemotherapy adherence scale; PAD = Peripheral artery disease; PDC = Proportion of days covered; PI = Principal investigator; PMC = Polymedication check; PQA = Pharmacy quality alliance; PTCA = Percutaneous Transluminal Coronary Angioplasty; RA = Rheumatoid arthritis; SOC = Standard of care; SMS = Short message system; SRM = Self-regulatory model; T2DM =Type 2 diabetes mellitus; UK = United Kingdom; USA = United States of America

Supplementary Table 4. Characteristics of included pre-post studies, NRCTs, and longitudinal studies.

**Pre-post studies**										
**First author**	**Year**	**Country**	**Patients (N)**	**Age**	**Disease or condition**	**Setting**	**Intervention**	**Providers**	**Follow-up**	**Adherence measurement tool**
Tahghighi et al.	2023	Iran	62	≥60 as per inclusion criteria	Bipolar disorder	Secondary care	A motivational-educational program with five components delivered in four sessions, covering medication review, side effects, adherence, and relapse prevention.	Research staff	2 months	MMAI
Balli et al.	2021	Turkey	94	Mean ± SD 80.6 ± 6.8	Dementia	Primary care	A clinical pharmacist assessed adherence, provided verbal information, and corrected misconceptions using the DKAT2 tool.	Pharmacists	4 months	MGLS
González-Bueno et al.	2021	Spain	77	≥65 as per inclusion criteria	Multimorbidity (≥2 chronic conditions) and polypharmacy (≥5 drugs)	Tertiary care	The intervention followed the PCP model, including patient assessment, medication optimization, reassessment of high-risk drugs, and an individualized plan.	Physicians and pharmacists	6 months	PDC

							Multidose systems were recommended for high-risk non-adherent patients.			
Vieira et al.	2021	Brazil	32	≥60 as per inclusion criteria	Hypertension	Primary care	Patients received the Supermed device, a monthly pill organizer with an alarm and electronic tracking. Medications were pre-packaged with labels, and packaging occurred monthly with patient involvement.	None	6 months	MMAS-4
Alkhoshaiban et al.	2019	Saudi Arabia	102	≥60 as per inclusion criteria	T2DM	Secondary care	The program aimed to enhance pharmacists' T2DM knowledge with 10 hours of training, including modules, lectures, demonstrations, and hands-on experience in diabetes clinics, along with posters and brochures	Research staff	8 months	MMAS-8
Lee S et al.	2018	USA	687	≥65 as per inclusion criteria	Multimorbidity (≥1 chronic condition)	Primary care	The intervention consisted of six 2.5-hour sessions over six weeks, covering topics like problem-solving, exercise, medication use, communication, nutrition, and new treatment options.	Research staff	12 months	MMAS-4
Doggrell et al.	2017	Australia	15	≥65 as per inclusion criteria	/	Primary care	AdherenceCheck assessed medication adherence, provided guidance, and created a personalized action plan, which was discussed and coordinated with the primary care physician within two weeks.	Pharmacists	6 months	MMAS-4
Chen et al.	2016	Taiwan	152	Mean ± SD 64.7 ± 9.7	Hypertension, hyperglycemia, or hyperlipidemia with polypharmacy (≥5 drugs)	Secondary care	Patients completed a pre-test, had medications reviewed, received education, and took a post-test. After three months, a follow-up call was made, and pillboxes were provided for adherence support.	Pharmacists	12 months	MPR
Mertens et al.	2016	Germany	24	≥60 as per inclusion criteria	CHD	Secondary care	The intervention included an initial phase without support, followed by 28 days using the "Medication Plan" app, and then a comparative phase with a paper diary for medication and blood pressure recording.	None	2 months	iPad; Paper Journal; Questionnaire

Casula et al.	2015	Italy	10,621 (5,833 in the PRE-i group, 4,788 in the POST-i group)	Mean ± SD PRE-i: 63.8 ± 12.0 POST-i: 63.0 ± 12.4	Initiating statin therapy	Primary care	The intervention had two components: a document with information on dyslipidemia, statins, and adherence, and aggregated data on statin use and adherence in 2006, compared to LHU and district averages.	None	12 months	MPR
Hawkins et al.	2014	USA	27	Mean ± SD 65.3 ± 8.2	HF with cognitive impairment	Secondary care	The intervention included an illustrated medication chart with drug images, doses, and descriptions, along with an optional pill organizer and alarm.	Physicians	4 months	Pill count
Stuurman-Bieze et al.	2014	The Netherla nds	937 (495 I, 442 C)	Mean ± SD I: 67.0 ± 13.9 C: 67.0 ± 15.2	Osteoporosis	Primary care	The MeMO intervention involved counseling at the first two osteoporosis dispensations, followed by quarterly adherence monitoring and interventions for missed refills after the third dispensing.	Community pharmacists	12 months	PDC
Kamimura et al.	2012	Japan	18	≥65 as per inclusion criteria	Cognitive impairment	Primary care	The intervention used a personalized automatic pill dispenser with reminders. Participants were assessed on its use, and caregivers monitored it during the first week.	None	3 months	SAMR
Zhang et al.	2010	USA	20,889	≥65 as per inclusion criteria	Hyperlipidemia, hypertension, and/or DM	Primary care	Before Part D, the intervention groups had varying drug coverage. After its introduction, all gained Part D plans, with enhanced benefits like no deductible, fixed co-pays, and optional generic coverage in the gap for a higher premium.	None	24 months	MPR
Insel and Cole	2005	USA	27	≥67 as per inclusion criteria	Hypertension, hyperlipidemia or arthritis	Primary care	The intervention used personalized reminders, memory aids, and strategic medication placement to support adherence, adapting to participants' routines and environments.	Research staff	2 months	Number of supplied days
Raynor et al.	2000	UK	143 pre, 125 post	≥65 as per inclusion criteria	Polypharmacy (≥4 drugs)	Primary care	The intervention included two home visits: one for medication review and action plan development, and another for a revised supply and regimen explanation, using a	Pharmacists	2 months	Questionnaire

							structured interview and self-assessment tool.			
**Non-randomized controlled trials (NRCTs)**										
**First author**	**Year**	**Country**	**Patients (N)**	**Age**	**Disease or condition**	**Setting**	**Intervention**	**Providers**	**Follow-up**	**Adherence measurement**
Son et al.	South Korea	2019	5,370	Range 65-84 as per inclusion criteria	Hypertension	Primary care	The intervention improved adherence through an awareness campaign, early diagnosis education, and hypertension control, with reminders and health education.	Research staff	48 months	Combination: DPP; DDPP
Brennan et al.	USA	2012	29,247 (5,123 I, 24.124 C)	Mean I: 64.1 C: 62.9	DM	Primary care	Pharmacists counseled patients on adherence, new therapies, and delayed refills, with follow-up calls and communication with doctors.	Pharmacists	18 months	Monthly change in the number of days supplied
Bilotta et al.	Italy	2011	108 (54 I, 54 C)	≥65 as per inclusion criteria	Polypharmacy (≥1 daily drug)	Secondary care	The intervention involved patients or caregivers transcribing the medication regimen under the doctor's supervision. The control group received an oral review and written instructions.	Physicians	1 month	Semi-structured interview
Smith GE et al.	2007	USA	14	Mean ± SD Video: 79.8 ± 11.4 Phone: 81.9 ± 11.0 Control: 85.5 ± 6.6	Dementia or mild cognitive impairment	Primary care	The intervention included daily video calls from nursing assistants to verify medication intake in one arm, and daily phone calls in the other. The control group received standard care with only data collection.	Nurses	6 months	Pill count
Kogos et al.	2004	USA	30 (20 I, 10 C)	≥65 as per inclusion criteria	Being non-adherent	Primary care	A 5-week support group focused on a personalized adherence contract, feedback, lifestyle education, social support, and daily medication tracking.	Research staff	1 month	Pill count
Schectman et al.	2004	USA	1,472 (340 I, 1,132 C)	Mean ± SD 61.6 ± 11.0	DM	Primary care	Doctors received feedback reports on medication adherence and a 30-minute educational session covering adherence assessment, barriers, and intervention strategies.	Research staff	6 months	Prescription refill history

Finley et al.	2002	USA	190 (61 I, 129 C)	Mean ± SD<br>I: 61.1 ± 16.2<br>C: 59.9 ± 15.9	Depression	Primary care	Pharmacists conducted an initial interview, followed by follow-ups at weeks 6 and 24, and regular phone check-ins for side effects and adherence. Adjustments were made with primary care providers' approval.	Pharmacists	6 months	MPR
Wolfe and Schirm	1992	USA	38 (18 I, 20 C)	≥65 as per inclusion criteria	Hospitalization (≥24 hours)	Tertiary care	The experimental group received nursing counseling and a Medication Fact Sheet with detailed drug information. The control group received standard care without counseling or MFS.	Nurses	1 month	MCRS
Leirer et al.	1991	USA	16 (8 I, 8 C)	Mean<br>70.9	Not being on polypharmacy (<2 drugs)	Primary care	The intervention used TeleMinder, a telephone reminder system with pre-recorded messages about medication intake, and portable barcode scanners for participants to track adherence by scanning medication barcodes.	None	1 month	Barcode scanner
**Longitudinal studies**										
**First author**	**Year**	**Country**	**Patients (N)**	**Age**	**Disease or condition**	**Setting**	**Intervention**	**Providers**	**Follow-up**	**Adherence measurement**
Smith-Ray et al.	2024	USA	4,000,946 (1,271,259 I, 2,729,687 C)	Mean I<br>2020: 70.1<br>2021: 71.0<br>2022: 71.7<br>Mean C<br>2020: 73.3<br>2021: 73.5<br>2022: 73.6	T2DM, hypertension or hyperlipidemia; Being on ≥2 drugs	Primary care	Machine learning was used to identify patients at risk of non-adherence. Pharmacists provided multichannel interventions (in-person, phone, digital) at the first prescription refill, addressing barriers and offering personalized support, including follow-up calls.	Pharmacists	24 months	PDC
Liu et al.	2022	China	106 (56 I, 50 C)	Mean ± SD<br>I: 69.5 ± 6.5<br>C: 68.8 ± 5.5	DM	Secondary care	Routine care vs. intensive personalized care with education, online support, home visits, and monitoring.	Nurses	6 months	On-time medication assumption
Guerard et al.	2018	USA	291,326 member-month observations	Mean ± SD<br>68.5 ± 9.2	T2DM	Primary care	CWA involved medication review and adherence education, conducted at home, care centers, or physician offices.	Nurses	60 months	Binary indicator

Desteghe et al.	2017	Belgium	13	Mean ± SD 69.2 ± 3.7	AF	Secondary care	The Health Buddies app linked AF patients and grandchildren via a virtual contract, featuring daily challenges, medication reminders, provider communication, education, and rewards over 90 days.	None	3 months	MMAS-8; App usage; MEMS; Pill count
Lester et al.	2016	USA	10,936 (2,392 I, 8,544)	≥65 as per inclusion criteria	Hyperlipidemia, hypertension or T2DM	Primary care	An automatic refill program dispensing medications recurrently, up to a week before depletion, without patient requests, based on pickup history.	None	12 months	PDC
Bisharat et al.	2012	Israel	74 (33 I, 41 C)	Mean ± SD I: 65.3 ± 12.2 C: 72.7 ± 10.6	CHF	Secondary care	Pharmacist counseling (20–30 min) followed nursing interviews, stressing adherence, missed doses, and doctor contact for health changes. Discrepancies with nursing advice were reconciled. Controls received standard nursing discharge counseling.	Pharmacists	6 months	MPR
Foreman et al.	2012	USA	580 (290 I, 290 C)	Mean ± SD I: 64.8 ± 11.9 C: 64.7 ± 13.7	Chronic diseases	Primary care	An SMS program providing refill, renewal, transfer alerts, shipment updates, and medication reminders.	None	8 months	PDC

AF = Atrial fibrillation; CHD = Coronary heart disease; CWA = Comprehensive wellness assessment; DDPP =Dispensation days per prescription; DKAT2 = Dementia knowledge and attitude test 2; DPP = Dispensations per prescription; GP = General practitioner; HF = Heart failure; LHU = Local health unit; MCRS = Medication rating scale; MEMS = Medication event monitoring system; MGLS = Morisky-Green-Levine scale; MMAI = Morisky medication adherence inventory; MMAS-4 = Four-item Morisky medication adherence scale; MMAS-8 = Eight-item Morisky medication adherence scale; MPR = Medication possession ratio; PCP = Patient-centered prescription; PDC = Proportion of days covered; T2DM = Type 2 diabetes mellitus; SMS = Short message system; UK = United Kingdom; USA = United States of America

References of included studies

Aleem A, Amin F, Asim MH, Farooq N, Arshad S, Raziq M. Impact of pharmacist-led interventions in improving adherence to glaucoma medications in the geriatric population. Eur J Hosp Pharm. 2021;28: e191-e196.

Alkhoshaiban A, Hassan Y, Loganathan M, Alomary M, Morisky DE, Alawwad B. Type II Diabetic Patients' Satisfaction, Medication Adherence, and Glycemic Control after the Application of Pharmacist Counseling Program. Archives of Pharmacy Practice. 2019;10: 127-136.

Balli FN, Unsal P, Halil MG, Dogu BB, Cankurtaran M, Demirkan K. Effect of clinical pharmacists' interventions on dementia treatment adherence and caregivers' knowledge. Geriatrics & gerontology international. 2021;21: 506-511.

Basoglu S, Polat U. The Effect of Education and Monitoring via Tele-Nursing to Elderly Cancer Patients Using Oral Anticancer Agents on Self-efficacy and Medication Adherence: A Randomized Controlled Trial. Semin Oncol Nurs. 2024;40: 151692.

Bilotta C, Lucini A, Nicolini P, Vergani C. An easy intervention to improve short-term adherence to medications in community-dwelling older outpatients. A pilot non-randomised controlled trial. BMC Health Serv Res. 2011;11: 158.

Bisharat B, Hafi L, Baron-Epel O, Armaly Z, Bowirrat A. Pharmacist counseling to cardiac patients in Israel prior to discharge from hospital contribute to increasing patient's medication adherence closing gaps and improving outcomes. J Transl Med. 2012;10: 34.

Bouvy ML, Heerdink ER, Urquhart J, Grobbee DE, Hoes AW, Leufkens HG. Effect of a pharmacist-led intervention on diuretic compliance in heart failure patients: a randomized controlled study. J Card Fail. 2003;9: 404-411.

Brath H, Morak J, Kastenbauer Tet al. Mobile health (mHealth) based medication adherence measurement - a pilot trial using electronic blisters in diabetes patients. Br J Clin Pharmacol. 2013;76 Suppl 1: 47-55.

Brennan TA, Dollear TJ, Hu Met al. An integrated pharmacy-based program improved medication prescription and adherence rates in diabetes patients. Health Aff (Millwood). 2012;31: 120-129.

Calvert SB, Kramer JM, Anstrom KJ, Kaltenbach LA, Stafford JA, Allen LaPointe NM. Patient-focused intervention to improve long-term adherence to evidence-based medications: a randomized trial. Am Heart J. 2012;163: 657-665 e651.

Calvo E, Izquierdo S, Castillo Ret al. Can an individualized adherence education program delivered by nurses improve therapeutic adherence in elderly people with acute myocardial infarction?: A randomized controlled study. Int J Nurs Stud. 2021;120: 103975.

Casula M, Tragni E, Piccinelli Ret al. A simple informative intervention in primary care increases statin adherence. Eur J Clin Pharmacol. 2016;72: 227-234.

Chen CM, Kuo LN, Cheng KJet al. The effect of medication therapy management service combined with a national PharmaCloud system for polypharmacy patients. Comput Methods Programs Biomed. 2016;134: 109-119.

Choi YJ, Kim YT, Yi HS, Lee SY, Lee WY. Effects of Community-Based Interventions on Medication Adherence and Hospitalization for Elderly Patients with Type 2 Diabetes at Primary Care Clinics in South Korea. Int J Environ Res Public Health. 2021;18.

Chow EP, Hassali MA, Saleem F, Aljadhey H. Effects of pharmacist-led patient education on diabetes-related knowledge and medication adherence: A home-based study. Health Education Journal. 2015;75: 421-433.

Daliri S, Kooij MJ, Scholte Op Reimer WJMet al. Effects of a transitional care programme on medication adherence in an older cardiac population: A randomized clinical trial. Br J Clin Pharmacol. 2022;88: 965-982.

Delavar F, Pashaeypoor S, Negarandeh R. The effects of self-management education tailored to health literacy on medication adherence and blood pressure control among elderly people with primary hypertension: A randomized controlled trial. Patient Educ Couns. 2020;103: 336-342.

Desteghe L, Kluts K, Vijgen Jet al. The Health Buddies App as a Novel Tool to Improve Adherence and Knowledge in Atrial Fibrillation Patients: A Pilot Study. JMIR Mhealth Uhealth. 2017;5: e98.

Desteghe L, Vijgen J, Koopman Pet al. Telemonitoring-based feedback improves adherence to non-vitamin K antagonist oral anticoagulants intake in patients with atrial fibrillation. Eur Heart J. 2018;39: 1394-1403.

Doggrell SA. Pilot study, in a rental retirement village, of an "AdherenceCheck" on the management of medicines by the older-aged. International journal of clinical pharmacy. 2017;39: 443-449.

Dong X, Tsang CCS, Zhao Set al. Effects of the Medicare Part D comprehensive medication review on medication adherence among patients with Alzheimer's disease. Curr Med Res Opin. 2021;37: 1581-1588.

Eimer S, Mahmoodi-Shan GR, Abdollahi AA. The Effect of Self-Care Education on Adherence to Treatment in Elderly Patients with Heart Failure: A Randomized Clinical Trial. Iran J Nurs Midwifery Res. 2023;28: 610-615.

Elliott RA, Barber N, Clifford S, Horne R, Hartley E. The cost effectiveness of a telephone-based pharmacy advisory service to improve adherence to newly prescribed medicines. Pharm World Sci. 2008;30: 17-23.

Esposito L. The effects of medication education on adherence to medication regimens in an elderly population. J Adv Nurs. 1995;21: 935-943.

Eussen SR, van der Elst ME, Klungel OHet al. A pharmaceutical care program to improve adherence to statin therapy: a randomized controlled trial. Ann Pharmacother. 2010;44: 1905-1913.

Eyler R, Shvets K, Blakely ML. Motivational Interviewing to Increase Postdischarge Antibiotic Adherence in Older Adults with Pneumonia. Consult Pharm. 2016;31: 38-43.

Farmer A, Hardeman W, Hughes Det al. An explanatory randomised controlled trial of a nurse-led, consultation-based intervention to support patients with adherence to taking glucose lowering medication for type 2 diabetes. BMC family practice. 2012;13: 30.

Faulkner MA, Wadibia EC, Lucas BD, Hilleman DE. Impact of pharmacy counseling on compliance and effectiveness of combination lipid-lowering therapy in patients undergoing coronary artery revascularization: a randomized, controlled trial. Pharmacotherapy. 2000;20: 410-416.

Finley PR, Rens HR, Pont JTet al. Impact of a collaborative pharmacy practice model on the treatment of depression in primary care. Am J Health Syst Pharm. 2002;59: 1518-1526.

Fiscella R, Caplan E, Kamble P, Bunniran S, Uribe C, Chandwani H. The Effect of an Educational Intervention on Adherence to Intraocular Pressure-Lowering Medications in a Large Cohort of Older Adults with Glaucoma. J Manag Care Spec Pharm. 2018;24: 1284-1294.

Foreman KF, Stockl KM, Le LBet al. Impact of a text messaging pilot program on patient medication adherence. Clin Ther. 2012;34: 1084-1091.

Ganda K, Schaffer A, Pearson S, Seibel MJ. Compliance and persistence to oral bisphosphonate therapy following initiation within a secondary fracture prevention program: a randomised controlled trial of specialist vs. non-specialist management. Osteoporos Int. 2014;25: 1345-1355.

Goldstein CM, Gathright EC, Dolansky MAet al. Randomized controlled feasibility trial of two telemedicine medication reminder systems for older adults with heart failure. J Telemed Telecare. 2014;20: 293-299.
Gonnelli S, Caffarelli C, Rossi Set al. How the knowledge of fracture risk might influence adherence to oral therapy of osteoporosis in Italy: the ADEOST study. Aging clinical and experimental research. 2016;28: 459-468.
Gonzalez-Bueno J, Sevilla-Sanchez D, Puigoriol-Juvanteny E, Molist-Brunet N, Codina-Jane C, Espaulella-Panicot J. Improving medication adherence and effective prescribing through a patient-centered prescription model in patients with multimorbidity. Eur J Clin Pharmacol. 2022;78: 127-137.
Goswami NJ, Dekoven M, Kuznik Aet al. Impact of an integrated intervention program on atorvastatin adherence: a randomized controlled trial. Int J Gen Med. 2013;6: 647-655.
Grant RW, Devita NG, Singer DE, Meigs JB. Improving adherence and reducing medication discrepancies in patients with diabetes. Ann Pharmacother. 2003;37: 962-969.
Guerard B, Omachonu V, Perez B, Sen B. The Effectiveness of a Comprehensive Wellness Assessment on Medication Adherence in a Medicare Advantage Plan Diabetic Population. J Healthc Manag. 2018;63: 132-141.
Guo Y, Chen Y, Lane DA, Liu L, Wang Y, Lip GYH. Mobile Health Technology for Atrial Fibrillation Management Integrating Decision Support, Education, and Patient Involvement: mAF App Trial. Am J Med. 2017;130: 1388-1396 e1386.
Hadji P, Blettner M, Harbeck Net al. The Patient's Anastrozole Compliance to Therapy (PACT) Program: a randomized, in-practice study on the impact of a standardized information program on persistence and compliance to adjuvant endocrine therapy in postmenopausal women with early breast cancer. Ann Oncol. 2013;24: 1505-1512.
Haider I, Pond GR, Cameron Ret al. A structured oral chemotherapy teaching tool to improve adherence in adults with multiple myeloma: A pilot randomized controlled trial. J Geriatr Oncol. 2024;15: 101735.
Hawkins LA, Firek CJ. Testing a novel pictorial medication sheet to improve adherence in veterans with heart failure and cognitive impairment. Heart Lung. 2014;43: 486-493.
Hedegaard U, Kjeldsen LJ, Pottegard Aet al. Improving Medication Adherence in Patients with Hypertension: A Randomized Trial. Am J Med. 2015;128: 1351-1361.
Ho PM, Lambert-Kerzner A, Carey EPet al. Multifaceted intervention to improve medication adherence and secondary prevention measures after acute coronary

syndrome hospital discharge: a randomized clinical trial. JAMA internal medicine. 2014;174: 186-193.

Insel KC, Cole L. Individualizing memory strategies to improve medication adherence. Appl Nurs Res. 2005;18: 199-204.

Insel KC, Einstein GO, Morrow DG, Koerner KM, Hepworth JT. Multifaceted Prospective Memory Intervention to Improve Medication Adherence. J Am Geriatr Soc. 2016;64: 561-568.

Ivers NM, Schwalm JD, Bouck Zet al. Interventions supporting long term adherence and decreasing cardiovascular events after myocardial infarction (ISLAND): pragmatic randomised controlled trial. BMJ. 2020;369: m1731.

Jaimalai W, Panuthai S, Chintanawat R, Juntasopeepun P. Effect of Medagogy–Based Medication Literacy Enhancement on Medication Adherence Among Older Persons with Physical Multimorbidity: Randomized Controlled Trial. Pacific Rim International Journal of Nursing Research. 2023;28: 21-37.

Kamimura T, Ishiwata R, Inoue T. Medication reminder device for the elderly patients with mild cognitive impairment. Am J Alzheimers Dis Other Demen. 2012;27: 238-242.

Khosravi A, Ravari A, Mirzaei T, Gholamrezapour M. Effects of a Comprehensive Care Program on the Readmission Rate and Adherence to Treatment in Elderly Patients with Chronic Obstructive Pulmonary Disease. Tanaffos. 2020;19: 401-412.

Kogos SC. Support Groups and Treatment Adherence in a Geriatric Outpatient Clinic. Journal of Clinical Psychology in Medical Settings. 2004;11: 275-282.

Kolcu M, Ergun A. Effect of a nurse-led hypertension management program on quality of life, medication adherence and hypertension management in older adults: A randomized controlled trial. Geriatrics & gerontology international. 2020;20: 1182-1189.

Kooy MJ, van Wijk BL, Heerdink ER, de Boer A, Bouvy ML. Does the use of an electronic reminder device with or without counseling improve adherence to lipid-lowering treatment? The results of a randomized controlled trial. Front Pharmacol. 2013;4: 69.

Krass I, Taylor SJ, Smith C, Armour CL. Impact on medication use and adherence of Australian pharmacists' diabetes care services. J Am Pharm Assoc (2003). 2005;45: 33-40.

Kripalani S, Schmotzer B, Jacobson TA. Improving Medication Adherence through Graphically Enhanced Interventions in Coronary Heart Disease (IMAGE-CHD): a randomized controlled trial. J Gen Intern Med. 2012;27: 1609-1617.

Lee JK, Grace KA, Taylor AJ. Effect of a pharmacy care program on medication adherence and persistence, blood pressure, and low-density lipoprotein cholesterol: a randomized controlled trial. JAMA. 2006;296: 2563-2571.

Lee S, Jiang L, Dowdy D, Hong YA, Ory MG. Effects of the Chronic Disease Self-Management Program on medication adherence among older adults. Transl Behav Med. 2019;9: 380-388.

Leirer VO, Morrow DG, Tanke ED, Pariante GM. Elders' nonadherence: its assessment and medication reminding by voice mail. Gerontologist. 1991;31: 514-520.

Lester CA, Mott DA, Chui MA. The Influence of a Community Pharmacy Automatic Prescription Refill Program on Medicare Part D Adherence Metrics. J Manag Care Spec Pharm. 2016;22: 801-807.

Lin CY, Yaseri M, Pakpour AHet al. Can a Multifaceted Intervention Including Motivational Interviewing Improve Medication Adherence, Quality of Life, and Mortality Rates in Older Patients Undergoing Coronary Artery Bypass Surgery? A Multicenter, Randomized Controlled Trial with 18-Month Follow-Up. Drugs Aging. 2017;34: 143-156.

Lipton HL, Bird JA. The impact of clinical pharmacists' consultations on geriatric patients' compliance and medical care use: a randomized controlled trial. Gerontologist. 1994;34: 307-315.

Liu J, Shi X, Guo B. Influence of Information-Based Continuous Care on Disease Control and Treatment Compliance of Elderly Diabetic Patients. Evid Based Complement Alternat Med. 2022;2022: 4023123.

Lourenco LB, Rodrigues RC, Ciol MAet al. A randomized controlled trial of the effectiveness of planning strategies in the adherence to medication for coronary artery disease. J Adv Nurs. 2014;70: 1616-1628.

Lourens H, Woodward MC. Impact of a Medication Card on Compliance in Older People. Australian Journal on Ageing. 1994;13: 72-76.

Lowe CJ, Raynor DK, Courtney EA, Purvis J, Teale C. Effects of self medication programme on knowledge of drugs and compliance with treatment in elderly patients. BMJ. 1995;310: 1229-1231.

Maddison R, Jiang Y, Stewart Ret al. An Intervention to Improve Medication Adherence in People With Heart Disease (Text4HeartII): Randomized Controlled Trial. JMIR Mhealth Uhealth. 2021;9: e24952.

Mertens A, Brandl C, Miron-Shatz Tet al. A mobile application improves therapy-adherence rates in elderly patients undergoing rehabilitation: A crossover design study

comparing documentation via iPad with paper-based control. Medicine (Baltimore). 2016;95: e4446.

Messerli M, Blozik E, Vriends N, Hersberger KE. Impact of a community pharmacist-led medication review on medicines use in patients on polypharmacy--a prospective randomised controlled trial. BMC Health Serv Res. 2016;16: 145.

Mira JJ, Navarro I, Botella Fet al. A Spanish pillbox app for elderly patients taking multiple medications: randomized controlled trial. J Med Internet Res. 2014;16: e99.

Mohan A, Majd Z, Johnson MLet al. A Motivational Interviewing Intervention to Improve Adherence to ACEIs/ARBs among Nonadherent Older Adults with Comorbid Hypertension and Diabetes. Drugs Aging. 2023;40: 377-390.

Moral RR, Torres LA, Ortega LPet al. Effectiveness of motivational interviewing to improve therapeutic adherence in patients over 65 years old with chronic diseases: A cluster randomized clinical trial in primary care. Patient Educ Couns. 2015;98: 977-983.

Muir KW, Ventura A, Stinnett SS, Enfiedjian A, Allingham RR, Lee PP. The influence of health literacy level on an educational intervention to improve glaucoma medication adherence. Patient Educ Couns. 2012;87: 160-164.

Murray MD, Young J, Hoke Set al. Pharmacist intervention to improve medication adherence in heart failure: a randomized trial. Ann Intern Med. 2007;146: 714-725.

Nguyen T, Nguyen TH, Nguyen PTet al. Pharmacist-Led Intervention to Enhance Medication Adherence in Patients With Acute Coronary Syndrome in Vietnam: A Randomized Controlled Trial. Front Pharmacol. 2018;9: 656.

Oakley SW, t. A pilot study assessing the effectiveness of a decision aid on patient adherence with oral bisphosphonate medication. The pharmaceutical journal : official organ of the Pharmaceutical society of Great Britain. 2006;276: 536-538.

Obreli-Neto PR, Guidoni CM, de Oliveira Baldoni Aet al. Effect of a 36-month pharmaceutical care program on pharmacotherapy adherence in elderly diabetic and hypertensive patients. International journal of clinical pharmacy. 2011;33: 642-649.

O'Connor PJ, Schmittdiel JA, Pathak RDet al. Randomized trial of telephone outreach to improve medication adherence and metabolic control in adults with diabetes. Diabetes Care. 2014;37: 3317-3324.

Odegard PS, Christensen DB. MAP study: RCT of a medication adherence program for patients with type 2 diabetes. J Am Pharm Assoc (2003). 2012;52: 753-762.

Olesen C, Harbig P, Buus KM, Barat I, Damsgaard EM. Impact of pharmaceutical care on adherence, hospitalisations and mortality in elderly patients. International journal of clinical pharmacy. 2014;36: 163-171.

Ownby RL, Hertzog C, Czaja SJ. Tailored Information and Automated Reminding to Improve Medication Adherence in Spanish- and English-Speaking Elders Treated for Memory Impairment. Clin Gerontol. 2012;35.

Pandey A, Krumme AA, Patel T, Choudhry NK. The Impact of Text Messaging on Medication Adherence and Exercise Among Postmyocardial Infarction Patients: Randomized Controlled Pilot Trial. JMIR Mhealth Uhealth. 2017;5: e110.

Poorcheraghi H, Negarandeh R, Pashaeypoor S, Jorian J. Effect of using a mobile drug management application on medication adherence and hospital readmission among elderly patients with polypharmacy: a randomized controlled trial. BMC Health Serv Res. 2023;23: 1192.

Qvist I, Lindholt JS, Sogaard R, Lorentzen V, Hallas J, Frost L. Randomised trial of telephone counselling to improve participants' adherence to prescribed drugs in a vascular screening trial. Basic & clinical pharmacology & toxicology. 2020;127: 477-487.

Raj JP, Mathews B. Effect of behavioral intervention on medication adherence among elderly with select non-communicable diseases (ENDORSE): Pilot randomized controlled trial. Geriatrics & gerontology international. 2020;20: 1079-1084.

Ranjbar H, Sadeghi-Vazin K, Bakhshi M. The cost-effectiveness of peer education on medication adherence in the elderly with hypertension: a randomized controlled trial. BMC Public Health. 2024;24: 3268.

Raynor DK, Nicolson M, Nunney J, Petty D, Vail A, Davies L. The development and evaluation of an extended adherence support programme by community pharmacists for elderly patients at home. International Journal of Pharmacy Practice. 2000;8: 157-164.

Rich MW, Gray DB, Beckham V, Wittenberg C, Luther P. Effect of a multidisciplinary intervention on medication compliance in elderly patients with congestive heart failure. Am J Med. 1996;101: 270-276.

Rinfret S, Rodes-Cabau J, Bagur Ret al. Telephone contact to improve adherence to dual antiplatelet therapy after drug-eluting stent implantation. Heart. 2013;99: 562-569.

Rosen MI, Rigsby MO, Salahi JT, Ryan CE, Cramer JA. Electronic monitoring and counseling to improve medication adherence. Behav Res Ther. 2004;42: 409-422.

Roshandel M, Rakhshan M, Najafi Kalyani M. The Effect of Using Peer on Self-Care, Quality of Life, and Adherence in Elderly People with Coronary Artery Disease. TheScientificWorldJournal. 2021;2021: 4770721.

Ruppar TM. Randomized pilot study of a behavioral feedback intervention to improve medication adherence in older adults with hypertension. J Cardiovasc Nurs. 2010;25: 470-479.

Samajdar SS, Tripathi R, Mukherjee Set al. Improving Medication Adherence in Geriatric T2DM Patients: A Factorial Randomized Controlled Trial. Journal of Diabetology. 2024;15: 389-394.

Schectman JM, Schorling JB, Nadkarni MM, Voss JD. Can prescription refill feedback to physicians improve patient adherence? Am J Med Sci. 2004;327: 19-24.

Schneider PJ, Murphy JE, Pedersen CA. Impact of medication packaging on adherence and treatment outcomes in older ambulatory patients. J Am Pharm Assoc (2003). 2008;48: 58-63.

Schroeder K, Fahey T, Hollinghurst S, Peters TJ. Nurse-led adherence support in hypertension: a randomized controlled trial. Fam Pract. 2005;22: 144-151.

Schulz M, Griese-Mammen N, Anker SDet al. Pharmacy-based interdisciplinary intervention for patients with chronic heart failure: results of the PHARM-CHF randomized controlled trial. Eur J Heart Fail. 2019;21: 1012-1021.

Shim YW, Chua SS, Wong HC, Alwi S. Collaborative intervention between pharmacists and physicians on elderly patients: a randomized controlled trial. Ther Clin Risk Manag. 2018;14: 1115-1125.

Sirey JA, Banerjee S, Marino Pet al. Adherence to Depression Treatment in Primary Care: A Randomized Clinical Trial. JAMA Psychiatry. 2017;74: 1129-1135.

Sirey JA, Bruce ML, Kales HC. Improving antidepressant adherence and depression outcomes in primary care: the treatment initiation and participation (TIP) program. Am J Geriatr Psychiatry. 2010;18: 554-562.

Smith DH, Kramer JM, Perrin Net al. A randomized trial of direct-to-patient communication to enhance adherence to beta-blocker therapy following myocardial infarction. Arch Intern Med. 2008;168: 477-483; discussion 483; quiz 447.

Smith GE, Lunde AM, Hathaway JC, Vickers KS. Telehealth home monitoring of solitary persons with mild dementia. Am J Alzheimers Dis Other Demen. 2007;22: 20-26.

Smith-Ray R, Feng L, Singh Tet al. Pharmacists as clinical care partners: How a pharmacist-led intervention is associated with improved medication adherence in older adults with common chronic conditions. J Manag Care Spec Pharm. 2024;30: 345-351.

Solmaz T, Altay B. The role of training and medication reminder wristwatch in adherence to treatment in geriatric patients diagnosed with hypertension: A randomized controlled trial. Geriatrics & gerontology international. 2024;24: 1189-1195.

Solomon DK, Portner TS, Bass GEet al. Clinical and economic outcomes in the hypertension and COPD arms of a multicenter outcomes study. J Am Pharm Assoc (Wash). 1998;38: 574-585.

Son KJ, Son HR, Park B, Kim HJ, Kim CB. A Community-Based Intervention for Improving Medication Adherence for Elderly Patients with Hypertension in Korea. Int J Environ Res Public Health. 2019;16.

Sturgess IK, McElnay JC, Hughes CM, Crealey G. Community pharmacy based provision of pharmaceutical care to older patients. Pharm World Sci. 2003;25: 218-226.

Stuurman-Bieze AG, Hiddink EG, van Boven JF, Vegter S. Proactive pharmaceutical care interventions decrease patients' nonadherence to osteoporosis medication. Osteoporos Int. 2014;25: 1807-1812.

Sutema IAMP, Jaya MKA, Bakta IM. Medicine reminder to improve treatment compliance on geriatric patients with diabetic neuropathy at Sanglah Central Hospital, Bali-Indonesia. Bali Medical Journal. 2018;7.

Tahghighi H, Mortazavi H, Manteghi AA, Armat MR. The effect of comprehensive individual motivational-educational program on medication adherence in elderly patients with bipolar disorders: An experimental study. J Educ Health Promot. 2023;12: 70.

Tuzun S, Akyuz G, Eskiyurt Net al. Impact of the training on the compliance and persistence of weekly bisphosphonate treatment in postmenopausal osteoporosis: a randomized controlled study. Int J Med Sci. 2013;10: 1880-1887.

Tzikas A, Samaras A, Kartas Aet al. Motivational Interviewing to Support Oral AntiCoagulation adherence in patients with non-valvular Atrial Fibrillation (MISOAC-AF): a randomized clinical trial. Eur Heart J Cardiovasc Pharmacother. 2021;7: f63-f71.

van der Laan DM, Elders PJM, Boons C, Nijpels G, van Dijk L, Hugtenburg JG. Effectiveness of a Patient-Tailored, Pharmacist-Led Intervention Program to Enhance Adherence to Antihypertensive Medication: The CATI Study. Front Pharmacol. 2018;9: 1057.

Vieira LB, Reis AMM, Ramos CA, Reis TMD, Cassiani SHB. The use of an electronic medication organizer device with alarm to improve medication adherence of older adults with hypertension. Einstein (Sao Paulo). 2021;19: eAO6011.

Vinluan CM, Wittman D, Morisky D. Effect of pharmacist discharge counselling on medication adherence in elderly heart failure patients: a pilot study. Journal of Pharmaceutical Health Services Research. 2015;6: 103-110.

Vollmer WM, Owen-Smith AA, Tom JOet al. Improving adherence to cardiovascular disease medications with information technology. Am J Manag Care. 2014;20: SP502-510.

Volume CI, Farris KB, Kassam R, Cox CE, Cave A. Pharmaceutical care research and education project: patient outcomes. J Am Pharm Assoc (Wash). 2001;41: 411-420.

Wald DS, Bestwick JP, Raiman L, Brendell R, Wald NJ. Randomised trial of text messaging on adherence to cardiovascular preventive treatment (INTERACT trial). PloS one. 2014;9: e114268.

Wang L, Zhao Y, Han Let al. Pharmacist-Led Management Model and Medication Adherence Among Patients With Chronic Heart Failure: A Randomized Clinical Trial. JAMA Netw Open. 2024;7: e2453976.

Wang Y, Liu F, Wang Q. Effects of dual-sufficiency modified nursing care on treatment compliance and adverse cardiovascular events in elderly patients with coronary heart disease after interventional surgery. Minerva Med. 2024.

Williams A, Manias E, Walker R. Interventions to improve medication adherence in people with multiple chronic conditions: a systematic review. J Adv Nurs. 2008;63: 132-143.

Wolfe SC, Schirm V. Medication counseling for the elderly: effects on knowledge and compliance after hospital discharge. Geriatr Nurs. 1992;13: 134-138.

Wong ZS, Siy B, Da Silva Lopes K, Georgiou A. Improving Patients' Medication Adherence and Outcomes in Nonhospital Settings Through eHealth: Systematic Review of Randomized Controlled Trials. J Med Internet Res. 2020;22: e17015.

Wu JR, Mark B, Knafl GJ, Dunbar SB, Chang PP, DeWalt DA. A multi-component, family-focused and literacy-sensitive intervention to improve medication adherence in patients with heart failure-A randomized controlled trial. Heart Lung. 2019;48: 507-514.

Wu Q, Zhang D, Zhao Qet al. Effects of transitional health management on adherence and prognosis in elderly patients with acute myocardial infarction in percutaneous coronary intervention: A cluster randomized controlled trial. PloS one. 2019;14: e0217535.

Yang C, Lee DTF, Wang X, Chair SY. Effects of a nurse-led medication self-management intervention on medication adherence and health outcomes in older people with multimorbidity: A randomised controlled trial. Int J Nurs Stud. 2022;134: 104314.

Yoon M, Lee JH, Kim ICet al. Smartphone App for Improving Self-Awareness of Adherence to Edoxaban Treatment in Patients With Atrial Fibrillation (ADHERE-App Trial): Randomized Controlled Trial. J Med Internet Res. 2024;26: e65010.

Zarate-Bravo E, Garcia-Vazquez JP, Torres-Cervantes Eet al. Supporting the Medication Adherence of Older Mexican Adults Through External Cues Provided With Ambient Displays: Feasibility Randomized Controlled Trial. JMIR Mhealth Uhealth. 2020;8: e14680.

Zhang Y, Lave JR, Donohue JM, Fischer MA, Chernew ME, Newhouse JP. The impact of Medicare Part D on medication adherence among older adults enrolled in Medicare-Advantage products. Med Care. 2010;48: 409-417.

Supplementary Figure 1. Geographical distribution of studies by country

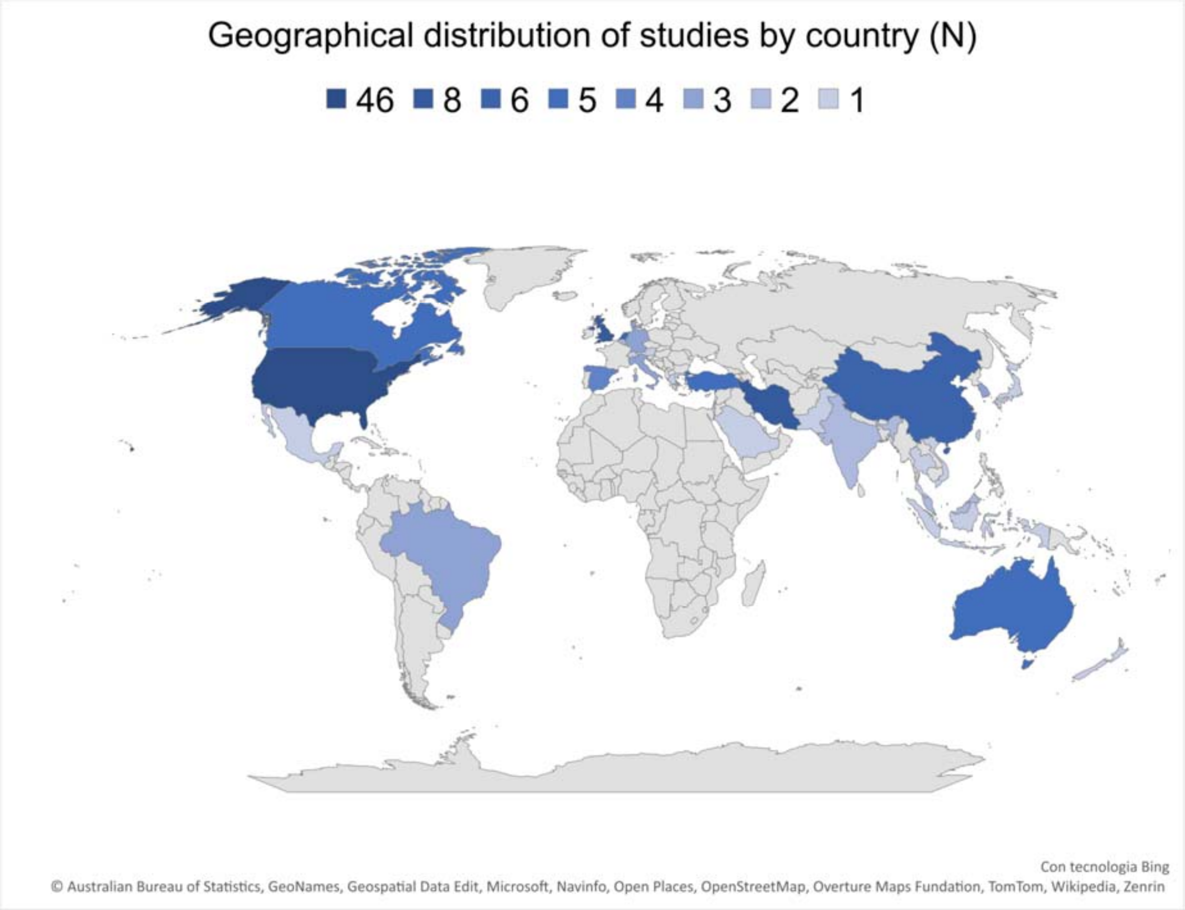

Supplementary Figure 2. Study distribution by type of intervention

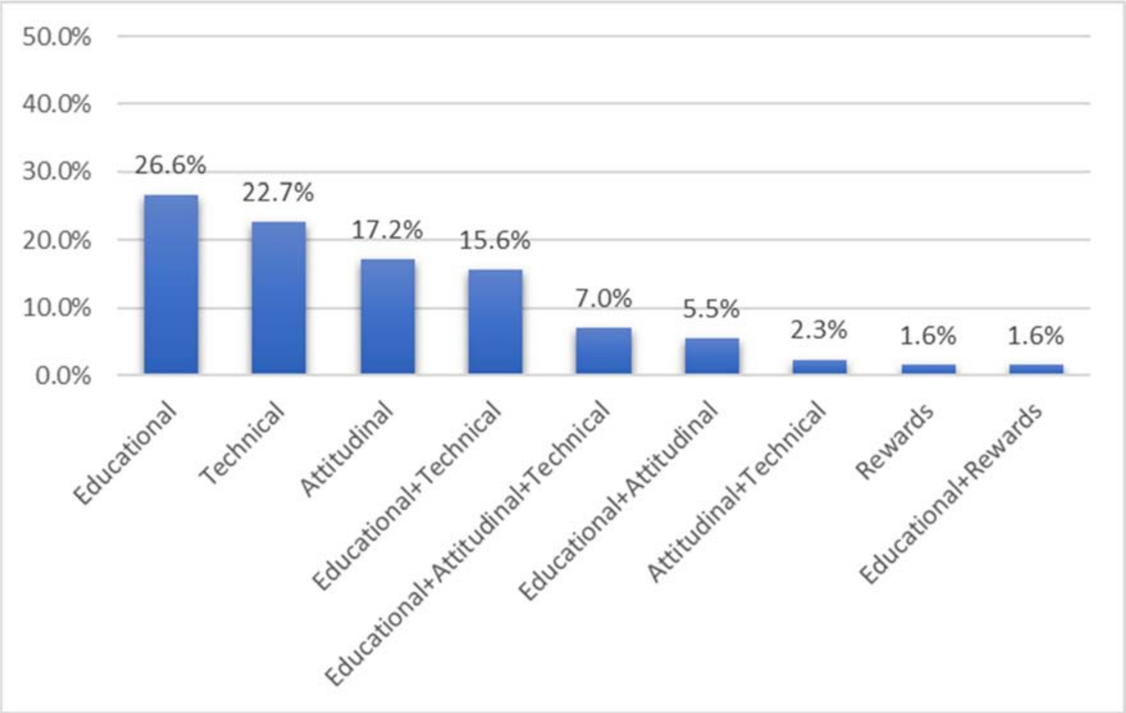

Supplementary Figure 3. Distribution of type of intervention based on health care setting

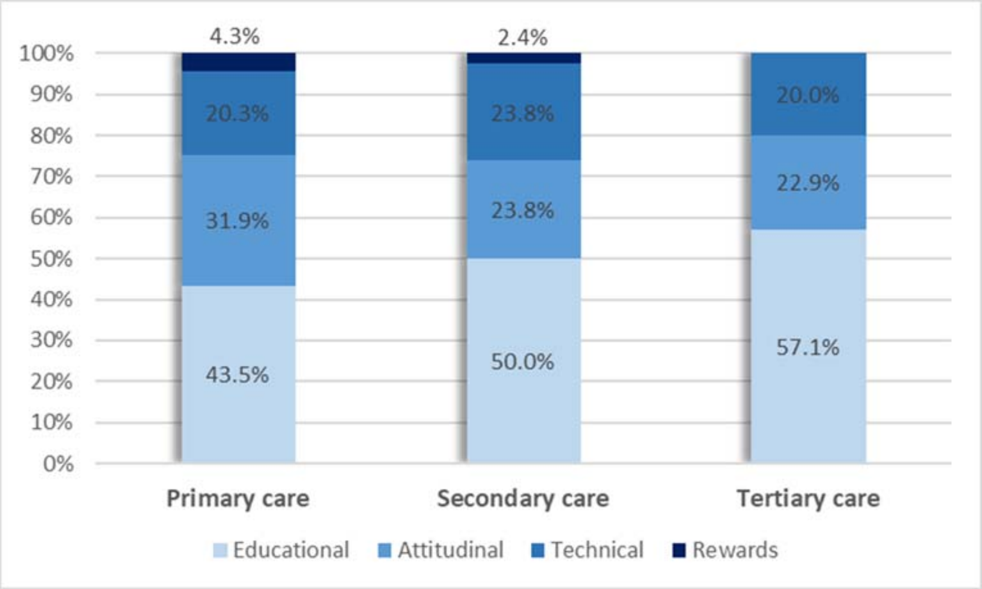

Supplementary Figure 4. Study distribution by intervention mediator

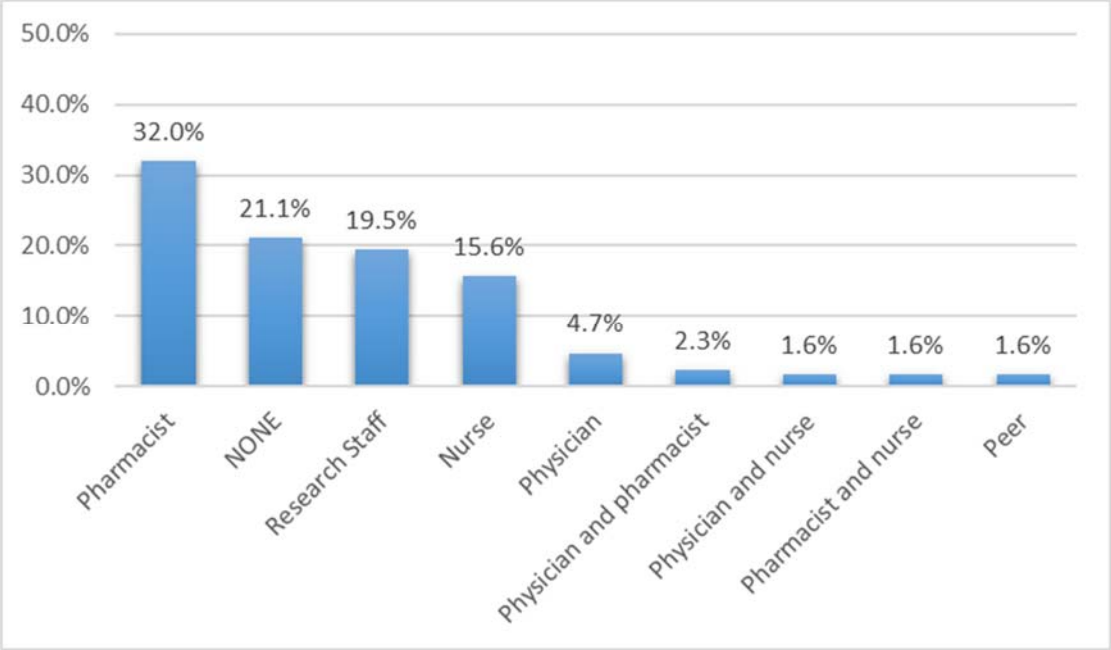

Supplementary Figure 5. Distribution of type of intervention based on mediator

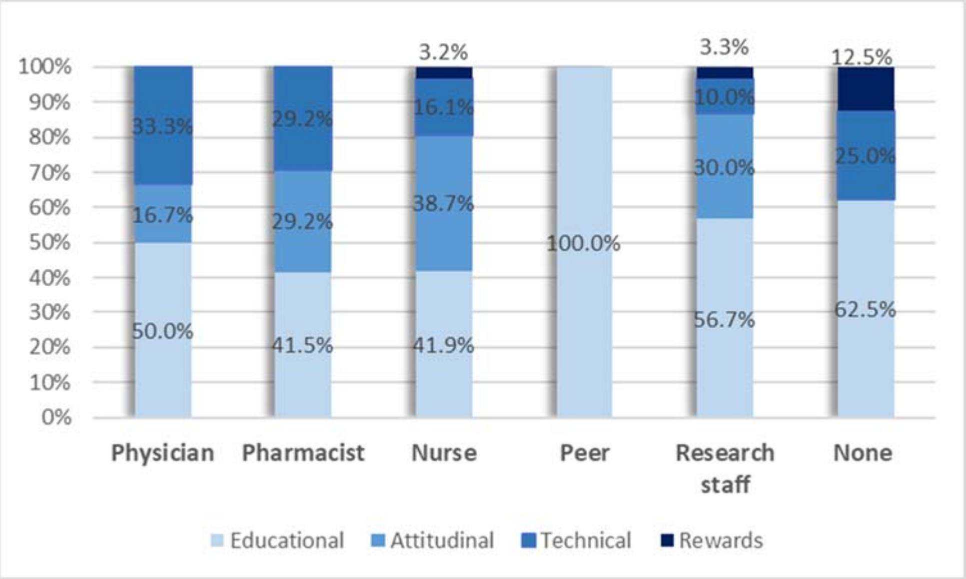

Supplementary Figure 6. Use of mediator based on healthcare setting

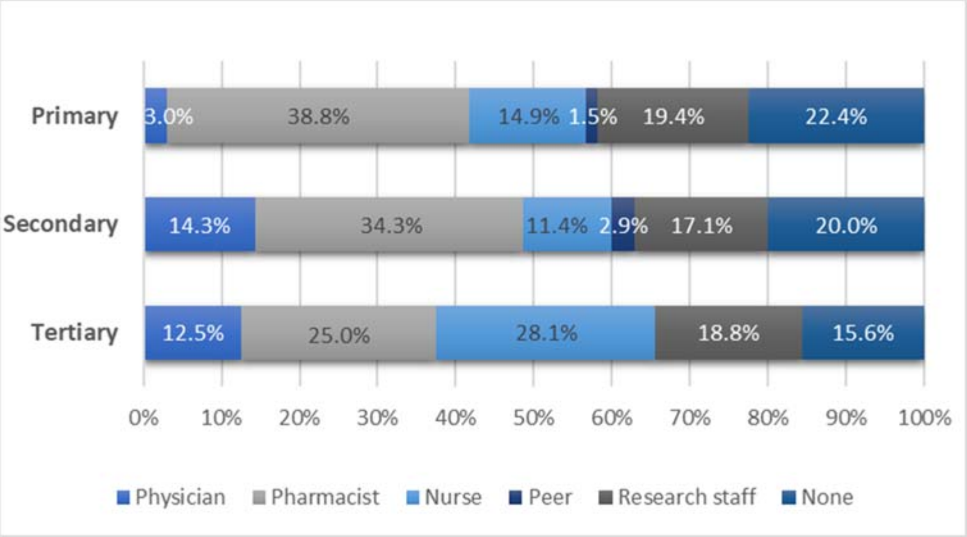

Supplementary Figure 7. Results of quality evaluation using RoBIAS tool – Summary plot

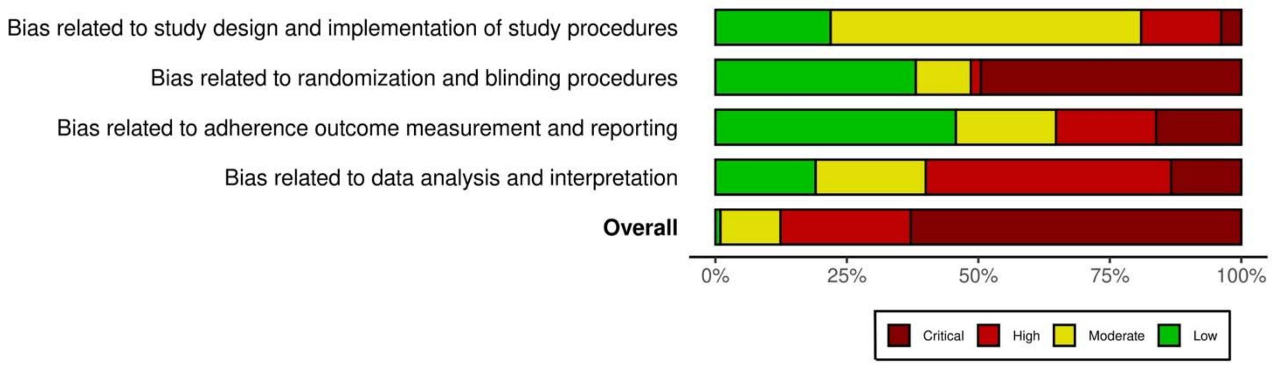

Supplementary Figure 8. Results of quality evaluation using RoBIAS tool – Traffic light

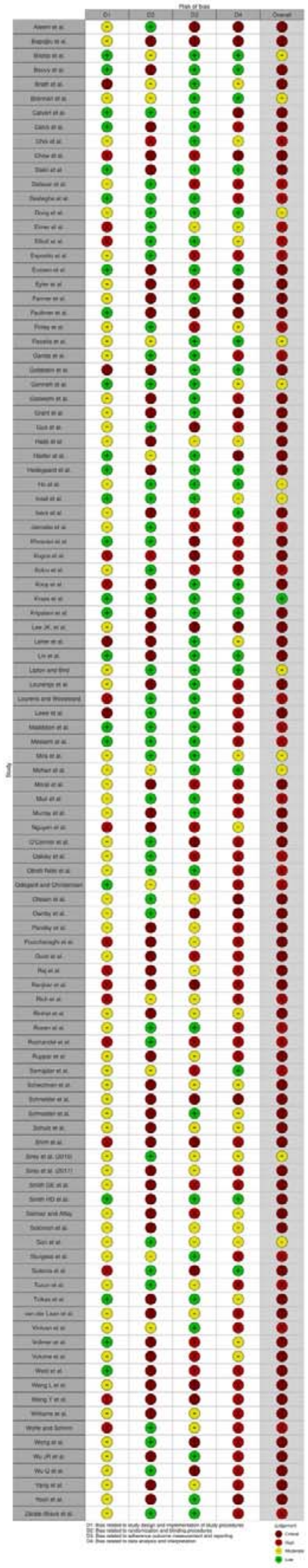

Supplementary Figure 9. Results of quality evaluation using RoBOAS tool – Summary plot

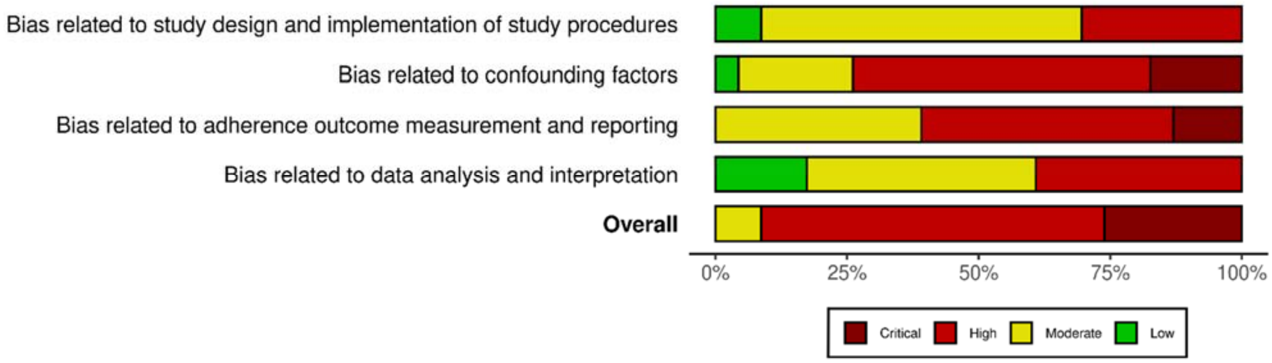

Supplementary Figure 10. Results of quality evaluation using RoBOAS tool – Traffic light

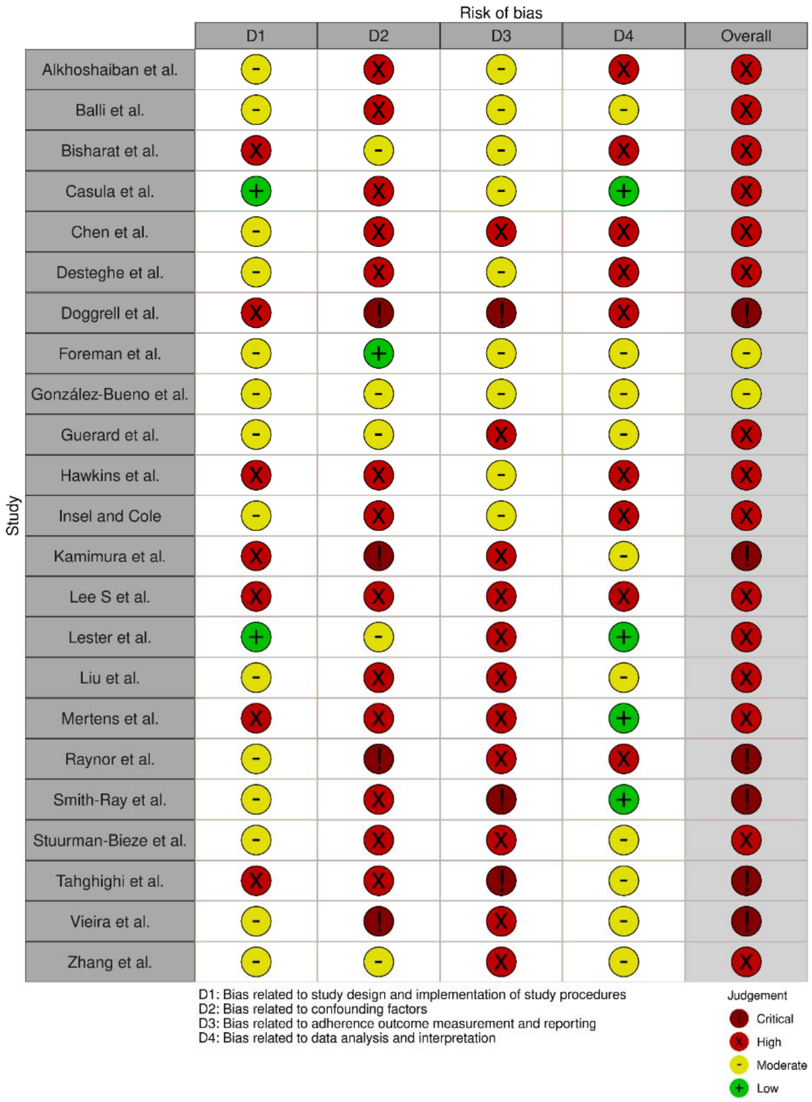

Supplement: Supplementary file 1 — Data S1: Supporting Information. [file JGS-74-479-s002.pdf]
