# Supplementary material for: Enhancing Medication Adherence in Older Adults: A Systematic Review of Evidence‐Based Strategies
Source: J Am Geriatr Soc. 2025 Dec 30;74(2):479–87. doi: 10.1111/jgs.70257 (PMC12911538; doi:10.1111/jgs.70257)
Supplement: Supplementary file 2 — Data S2: Supporting Information. [file JGS-74-479-s001.docx]

**References of article included in the systematic review**

S1. Basoglu S, Polat U. The Effect of Education and Monitoring via Tele-Nursing to Elderly Cancer Patients Using Oral Anticancer Agents on Self-efficacy and Medication Adherence: A Randomized Controlled Trial. Semin Oncol Nurs. 2024;40: 151692.

S2. Haider I, Pond GR, Cameron Ret al. A structured oral chemotherapy teaching tool to improve adherence in adults with multiple myeloma: A pilot randomized controlled trial. J Geriatr Oncol. 2024;15: 101735.

S3. Ranjbar H, Sadeghi-Vazin K, Bakhshi M. The cost-effectiveness of peer education on medication adherence in the elderly with hypertension: a randomized controlled trial. BMC Public Health. 2024;24: 3268.

S4. Samajdar SS, Tripathi R, Mukherjee Set al. Improving Medication Adherence in Geriatric T2DM Patients: A Factorial Randomized Controlled Trial. Journal of Diabetology. 2024;15: 389-394.

S5. Wang L, Zhao Y, Han Let al. Pharmacist-Led Management Model and Medication Adherence Among Patients With Chronic Heart Failure: A Randomized Clinical Trial. JAMA Netw Open. 2024;7: e2453976.

S6. Wang Y, Liu F, Wang Q. Effects of dual-sufficiency modified nursing care on treatment compliance and adverse cardiovascular events in elderly patients with coronary heart disease after interventional surgery. Minerva Med. 2024.

S7. Yoon M, Lee JH, Kim ICet al. Smartphone App for Improving Self-Awareness of Adherence to Edoxaban Treatment in Patients With Atrial Fibrillation (ADHERE-App Trial): Randomized Controlled Trial. J Med Internet Res. 2024;26: e65010.

S8. Eimer S, Mahmoodi-Shan GR, Abdollahi AA. The Effect of Self-Care Education on Adherence to Treatment in Elderly Patients with Heart Failure: A Randomized Clinical Trial. Iran J Nurs Midwifery Res. 2023;28: 610-615.

S9. Aleem A, Amin F, Asim MH, Farooq N, Arshad S, Raziq M. Impact of pharmacist-led interventions in improving adherence to glaucoma medications in the geriatric population. Eur J Hosp Pharm. 2021;28: e191-e196.

S10. Calvo E, Izquierdo S, Castillo Ret al. Can an individualized adherence education program delivered by nurses improve therapeutic adherence in elderly people with acute myocardial infarction?: A randomized controlled study. Int J Nurs Stud. 2021;120: 103975.

S11. Choi YJ, Kim YT, Yi HS, Lee SY, Lee WY. Effects of Community-Based Interventions on Medication Adherence and Hospitalization for Elderly Patients with Type 2 Diabetes at Primary Care Clinics in South Korea. Int J Environ Res Public Health. 2021;18.

S12. Maddison R, Jiang Y, Stewart Ret al. An Intervention to Improve Medication Adherence in People With Heart Disease (Text4HeartII): Randomized Controlled Trial. JMIR Mhealth Uhealth. 2021;9: e24952.

S13. Roshandel M, Rakhshan M, Najafi Kalyani M. The Effect of Using Peer on Self-Care, Quality of Life, and Adherence in Elderly People with Coronary Artery Disease. TheScientificWorldJournal. 2021;2021: 4770721.

S14. Tzikas A, Samaras A, Kartas Aet al. Motivational Interviewing to Support Oral AntiCoagulation adherence in patients with non-valvular Atrial Fibrillation (MISOAC-AF): a randomized clinical trial. Eur Heart J Cardiovasc Pharmacother. 2021;7: f63-f71.

S15. Delavar F, Pashaeypoor S, Negarandeh R. The effects of self-management education tailored to health literacy on medication adherence and blood pressure control among elderly people with primary hypertension: A randomized controlled trial. Patient Educ Couns. 2020;103: 336-342.

S16. Ivers NM, Schwalm JD, Bouck Zet al. Interventions supporting long term adherence and decreasing cardiovascular events after myocardial infarction (ISLAND): pragmatic randomised controlled trial. BMJ. 2020;369: m1731.

S17. Khosravi A, Ravari A, Mirzaei T, Gholamrezapour M. Effects of a Comprehensive Care Program on the Readmission Rate and Adherence to Treatment in Elderly Patients with Chronic Obstructive Pulmonary Disease. Tanaffos. 2020;19: 401-412.

S18. Kolcu M, Ergun A. Effect of a nurse-led hypertension management program on quality of life, medication adherence and hypertension management in older adults: A randomized controlled trial. Geriatrics & gerontology international. 2020;20: 1182-1189.

S19. Schulz M, Griese-Mammen N, Anker SDet al. Pharmacy-based interdisciplinary intervention for patients with chronic heart failure: results of the PHARM-CHF randomized controlled trial. Eur J Heart Fail. 2019;21: 1012-1021.

S20. Wu JR, Mark B, Knafl GJ, Dunbar SB, Chang PP, DeWalt DA. A multi-component, family-focused and literacy-sensitive intervention to improve medication adherence in patients with heart failure-A randomized controlled trial. Heart Lung. 2019;48: 507-514.

S21. Wu Q, Zhang D, Zhao Qet al. Effects of transitional health management on adherence and prognosis in elderly patients with acute myocardial infarction in percutaneous coronary intervention: A cluster randomized controlled trial. PloS one. 2019;14: e0217535.

S22. Desteghe L, Vijgen J, Koopman Pet al. Telemonitoring-based feedback improves adherence to non-vitamin K antagonist oral anticoagulants intake in patients with atrial fibrillation. Eur Heart J. 2018;39: 1394-1403.

S23. Fiscella R, Caplan E, Kamble P, Bunniran S, Uribe C, Chandwani H. The Effect of an Educational Intervention on Adherence to Intraocular Pressure-Lowering Medications in a Large Cohort of Older Adults with Glaucoma. J Manag Care Spec Pharm. 2018;24: 1284-1294.

S24. Nguyen T, Nguyen TH, Nguyen PTet al. Pharmacist-Led Intervention to Enhance Medication Adherence in Patients With Acute Coronary Syndrome in Vietnam: A Randomized Controlled Trial. Front Pharmacol. 2018;9: 656.

S25. Sutema IAMP, Jaya MKA, Bakta IM. Medicine reminder to improve treatment compliance on geriatric patients with diabetic neuropathy at Sanglah Central Hospital, Bali-Indonesia. Bali Medical Journal. 2018;7.

S26. van der Laan DM, Elders PJM, Boons C, Nijpels G, van Dijk L, Hugtenburg JG. Effectiveness of a Patient-Tailored, Pharmacist-Led Intervention Program to Enhance Adherence to Antihypertensive Medication: The CATI Study. Front Pharmacol. 2018;9: 1057.

S27. Guo Y, Chen Y, Lane DA, Liu L, Wang Y, Lip GYH. Mobile Health Technology for Atrial Fibrillation Management Integrating Decision Support, Education, and Patient Involvement: mAF App Trial. Am J Med. 2017;130: 1388-1396 e1386.

S28. Lin CY, Yaseri M, Pakpour AHet al. Can a Multifaceted Intervention Including Motivational Interviewing Improve Medication Adherence, Quality of Life, and Mortality Rates in Older Patients Undergoing Coronary Artery Bypass Surgery? A Multicenter, Randomized Controlled Trial with 18-Month Follow-Up. Drugs Aging. 2017;34: 143-156.

S29. Pandey A, Krumme AA, Patel T, Choudhry NK. The Impact of Text Messaging on Medication Adherence and Exercise Among Postmyocardial Infarction Patients: Randomized Controlled Pilot Trial. JMIR Mhealth Uhealth. 2017;5: e110.

S30. Sirey JA, Banerjee S, Marino Pet al. Adherence to Depression Treatment in Primary Care: A Randomized Clinical Trial. JAMA Psychiatry. 2017;74: 1129-1135.

S31. Eyler R, Shvets K, Blakely ML. Motivational Interviewing to Increase Postdischarge Antibiotic Adherence in Older Adults with Pneumonia. Consult Pharm. 2016;31: 38-43.

S32. Gonnelli S, Caffarelli C, Rossi Set al. How the knowledge of fracture risk might influence adherence to oral therapy of osteoporosis in Italy: the ADEOST study. Aging clinical and experimental research. 2016;28: 459-468.

S33. Insel KC, Einstein GO, Morrow DG, Koerner KM, Hepworth JT. Multifaceted Prospective Memory Intervention to Improve Medication Adherence. J Am Geriatr Soc. 2016;64: 561-568.

S34. Chow EP, Hassali MA, Saleem F, Aljadhey H. Effects of pharmacist-led patient education on diabetes-related knowledge and medication adherence: A home-based study. Health Education Journal. 2015;75: 421-433.

S35. Vinluan CM, Wittman D, Morisky D. Effect of pharmacist discharge counselling on medication adherence in elderly heart failure patients: a pilot study. Journal of Pharmaceutical Health Services Research. 2015;6: 103-110.

S36. Ganda K, Schaffer A, Pearson S, Seibel MJ. Compliance and persistence to oral bisphosphonate therapy following initiation within a secondary fracture prevention program: a randomised controlled trial of specialist vs. non-specialist management. Osteoporos Int. 2014;25: 1345-1355.

S37. Goldstein CM, Gathright EC, Dolansky MAet al. Randomized controlled feasibility trial of two telemedicine medication reminder systems for older adults with heart failure. J Telemed Telecare. 2014;20: 293-299.

S38. Goswami NJ, Dekoven M, Kuznik Aet al. Impact of an integrated intervention program on atorvastatin adherence: a randomized controlled trial. Int J Gen Med. 2013;6: 647-655.

S39. Lourenco LB, Rodrigues RC, Ciol MAet al. A randomized controlled trial of the effectiveness of planning strategies in the adherence to medication for coronary artery disease. J Adv Nurs. 2014;70: 1616-1628.

S40. O'Connor PJ, Schmittdiel JA, Pathak RDet al. Randomized trial of telephone outreach to improve medication adherence and metabolic control in adults with diabetes. Diabetes Care. 2014;37: 3317-3324.

S41. Hadji P, Blettner M, Harbeck Net al. The Patient's Anastrozole Compliance to Therapy (PACT) Program: a randomized, in-practice study on the impact of a standardized information program on persistence and compliance to adjuvant endocrine therapy in postmenopausal women with early breast cancer. Ann Oncol. 2013;24: 1505-1512.

S42. Kooy MJ, van Wijk BL, Heerdink ER, de Boer A, Bouvy ML. Does the use of an electronic reminder device with or without counseling improve adherence to lipid-lowering treatment? The results of a randomized controlled trial. Front Pharmacol. 2013;4: 69.

S43. Rinfret S, Rodes-Cabau J, Bagur Ret al. Telephone contact to improve adherence to dual antiplatelet therapy after drug-eluting stent implantation. Heart. 2013;99: 562-569.

S44. Tuzun S, Akyuz G, Eskiyurt Net al. Impact of the training on the compliance and persistence of weekly bisphosphonate treatment in postmenopausal osteoporosis: a randomized controlled study. Int J Med Sci. 2013;10: 1880-1887.

S45. Wong ZS, Siy B, Da Silva Lopes K, Georgiou A. Improving Patients' Medication Adherence and Outcomes in Nonhospital Settings Through eHealth: Systematic Review of Randomized Controlled Trials. J Med Internet Res. 2020;22: e17015.

S46. Calvert SB, Kramer JM, Anstrom KJ, Kaltenbach LA, Stafford JA, Allen LaPointe NM. Patient-focused intervention to improve long-term adherence to evidence-based medications: a randomized trial. Am Heart J. 2012;163: 657-665 e651.

S47. Farmer A, Hardeman W, Hughes Det al. An explanatory randomised controlled trial of a nurse-led, consultation-based intervention to support patients with adherence to taking glucose lowering medication for type 2 diabetes. BMC family practice. 2012;13: 30.

S48. Kripalani S, Schmotzer B, Jacobson TA. Improving Medication Adherence through Graphically Enhanced Interventions in Coronary Heart Disease (IMAGE-CHD): a randomized controlled trial. J Gen Intern Med. 2012;27: 1609-1617.

S49. Muir KW, Ventura A, Stinnett SS, Enfiedjian A, Allingham RR, Lee PP. The influence of health literacy level on an educational intervention to improve glaucoma medication adherence. Patient Educ Couns. 2012;87: 160-164.

S50. Odegard PS, Christensen DB. MAP study: RCT of a medication adherence program for patients with type 2 diabetes. J Am Pharm Assoc (2003). 2012;52: 753-762.

S51. Ownby RL, Hertzog C, Czaja SJ. Tailored Information and Automated Reminding to Improve Medication Adherence in Spanish- and English-Speaking Elders Treated for Memory Impairment. Clin Gerontol. 2012;35.

S52. Eussen SR, van der Elst ME, Klungel OHet al. A pharmaceutical care program to improve adherence to statin therapy: a randomized controlled trial. Ann Pharmacother. 2010;44: 1905-1913.

S53. Ruppar TM. Randomized pilot study of a behavioral feedback intervention to improve medication adherence in older adults with hypertension. J Cardiovasc Nurs. 2010;25: 470-479.

S54. Sirey JA, Bruce ML, Kales HC. Improving antidepressant adherence and depression outcomes in primary care: the treatment initiation and participation (TIP) program. Am J Geriatr Psychiatry. 2010;18: 554-562.

S55. Oakley SW, t. A pilot study assessing the effectiveness of a decision aid on patient adherence with oral bisphosphonate medication. The pharmaceutical journal : official organ of the Pharmaceutical society of Great Britain. 2006;276: 536-538.

S56. Schneider PJ, Murphy JE, Pedersen CA. Impact of medication packaging on adherence and treatment outcomes in older ambulatory patients. J Am Pharm Assoc (2003). 2008;48: 58-63.

S57. Smith DH, Kramer JM, Perrin Net al. A randomized trial of direct-to-patient communication to enhance adherence to beta-blocker therapy following myocardial infarction. Arch Intern Med. 2008;168: 477-483; discussion 483; quiz 447.

S58. Murray MD, Young J, Hoke Set al. Pharmacist intervention to improve medication adherence in heart failure: a randomized trial. Ann Intern Med. 2007;146: 714-725.

S59. Schroeder K, Fahey T, Hollinghurst S, Peters TJ. Nurse-led adherence support in hypertension: a randomized controlled trial. Fam Pract. 2005;22: 144-151.

S60. Rosen MI, Rigsby MO, Salahi JT, Ryan CE, Cramer JA. Electronic monitoring and counseling to improve medication adherence. Behav Res Ther. 2004;42: 409-422.

S61. Bouvy ML, Heerdink ER, Urquhart J, Grobbee DE, Hoes AW, Leufkens HG. Effect of a pharmacist-led intervention on diuretic compliance in heart failure patients: a randomized controlled study. J Card Fail. 2003;9: 404-411.

S62. Grant RW, Devita NG, Singer DE, Meigs JB. Improving adherence and reducing medication discrepancies in patients with diabetes. Ann Pharmacother. 2003;37: 962-969.

S63. Rich MW, Gray DB, Beckham V, Wittenberg C, Luther P. Effect of a multidisciplinary intervention on medication compliance in elderly patients with congestive heart failure. Am J Med. 1996;101: 270-276.

S64. Esposito L. The effects of medication education on adherence to medication regimens in an elderly population. J Adv Nurs. 1995;21: 935-943.

S65. Solmaz T, Altay B. The role of training and medication reminder wristwatch in adherence to treatment in geriatric patients diagnosed with hypertension: A randomized controlled trial. Geriatrics & gerontology international. 2024;24: 1189-1195.

S66. Jaimalai W, Panuthai S, Chintanawat R, Juntasopeepun P. Effect of Medagogy–Based Medication Literacy Enhancement on Medication Adherence Among Older Persons with Physical Multimorbidity: Randomized Controlled Trial. Pacific Rim International Journal of Nursing Research. 2023;28: 21-37.

S67. Mohan A, Majd Z, Johnson MLet al. A Motivational Interviewing Intervention to Improve Adherence to ACEIs/ARBs among Nonadherent Older Adults with Comorbid Hypertension and Diabetes. Drugs Aging. 2023;40: 377-390.

S68. Poorcheraghi H, Negarandeh R, Pashaeypoor S, Jorian J. Effect of using a mobile drug management application on medication adherence and hospital readmission among elderly patients with polypharmacy: a randomized controlled trial. BMC Health Serv Res. 2023;23: 1192.

S69. Daliri S, Kooij MJ, Scholte Op Reimer WJMet al. Effects of a transitional care programme on medication adherence in an older cardiac population: A randomized clinical trial. Br J Clin Pharmacol. 2022;88: 965-982.

S70. Yang C, Lee DTF, Wang X, Chair SY. Effects of a nurse-led medication self-management intervention on medication adherence and health outcomes in older people with multimorbidity: A randomised controlled trial. Int J Nurs Stud. 2022;134: 104314.

S71. Dong X, Tsang CCS, Zhao Set al. Effects of the Medicare Part D comprehensive medication review on medication adherence among patients with Alzheimer's disease. Curr Med Res Opin. 2021;37: 1581-1588.

S72. Qvist I, Lindholt JS, Sogaard R, Lorentzen V, Hallas J, Frost L. Randomised trial of telephone counselling to improve participants' adherence to prescribed drugs in a vascular screening trial. Basic & clinical pharmacology & toxicology. 2020;127: 477-487.

S73. Raj JP, Mathews B. Effect of behavioral intervention on medication adherence among elderly with select non-communicable diseases (ENDORSE): Pilot randomized controlled trial. Geriatrics & gerontology international. 2020;20: 1079-1084.

S74. Zarate-Bravo E, Garcia-Vazquez JP, Torres-Cervantes Eet al. Supporting the Medication Adherence of Older Mexican Adults Through External Cues Provided With Ambient Displays: Feasibility Randomized Controlled Trial. JMIR Mhealth Uhealth. 2020;8: e14680.

S75. Shim YW, Chua SS, Wong HC, Alwi S. Collaborative intervention between pharmacists and physicians on elderly patients: a randomized controlled trial. Ther Clin Risk Manag. 2018;14: 1115-1125.

S76. Messerli M, Blozik E, Vriends N, Hersberger KE. Impact of a community pharmacist-led medication review on medicines use in patients on polypharmacy--a prospective randomised controlled trial. BMC Health Serv Res. 2016;16: 145.

S77. Moral RR, Torres LA, Ortega LPet al. Effectiveness of motivational interviewing to improve therapeutic adherence in patients over 65 years old with chronic diseases: A cluster randomized clinical trial in primary care. Patient Educ Couns. 2015;98: 977-983.

S78. Hedegaard U, Kjeldsen LJ, Pottegard Aet al. Improving Medication Adherence in Patients with Hypertension: A Randomized Trial. Am J Med. 2015;128: 1351-1361.

S79. Mira JJ, Navarro I, Botella Fet al. A Spanish pillbox app for elderly patients taking multiple medications: randomized controlled trial. J Med Internet Res. 2014;16: e99.

S80. Vollmer WM, Owen-Smith AA, Tom JOet al. Improving adherence to cardiovascular disease medications with information technology. Am J Manag Care. 2014;20: SP502-510.

S81. Wald DS, Bestwick JP, Raiman L, Brendell R, Wald NJ. Randomised trial of text messaging on adherence to cardiovascular preventive treatment (INTERACT trial). PloS one. 2014;9: e114268.

S82. Brath H, Morak J, Kastenbauer Tet al. Mobile health (mHealth) based medication adherence measurement - a pilot trial using electronic blisters in diabetes patients. Br J Clin Pharmacol. 2013;76 Suppl 1: 47-55.

S83. Ho PM, Lambert-Kerzner A, Carey EPet al. Multifaceted intervention to improve medication adherence and secondary prevention measures after acute coronary syndrome hospital discharge: a randomized clinical trial. JAMA internal medicine. 2014;174: 186-193.

S84. Olesen C, Harbig P, Buus KM, Barat I, Damsgaard EM. Impact of pharmaceutical care on adherence, hospitalisations and mortality in elderly patients. International journal of clinical pharmacy. 2014;36: 163-171.

S85. Williams A, Manias E, Walker R. Interventions to improve medication adherence in people with multiple chronic conditions: a systematic review. J Adv Nurs. 2008;63: 132-143.

S86. Obreli-Neto PR, Guidoni CM, de Oliveira Baldoni Aet al. Effect of a 36-month pharmaceutical care program on pharmacotherapy adherence in elderly diabetic and hypertensive patients. International journal of clinical pharmacy. 2011;33: 642-649.

S87. Elliott RA, Barber N, Clifford S, Horne R, Hartley E. The cost effectiveness of a telephone-based pharmacy advisory service to improve adherence to newly prescribed medicines. Pharm World Sci. 2008;30: 17-23.

S88. Lee JK, Grace KA, Taylor AJ. Effect of a pharmacy care program on medication adherence and persistence, blood pressure, and low-density lipoprotein cholesterol: a randomized controlled trial. JAMA. 2006;296: 2563-2571.

S89. Krass I, Taylor SJ, Smith C, Armour CL. Impact on medication use and adherence of Australian pharmacists' diabetes care services. J Am Pharm Assoc (2003). 2005;45: 33-40.

S90. Sturgess IK, McElnay JC, Hughes CM, Crealey G. Community pharmacy based provision of pharmaceutical care to older patients. Pharm World Sci. 2003;25: 218-226.

S91. Volume CI, Farris KB, Kassam R, Cox CE, Cave A. Pharmaceutical care research and education project: patient outcomes. J Am Pharm Assoc (Wash). 2001;41: 411-420.

S92. Faulkner MA, Wadibia EC, Lucas BD, Hilleman DE. Impact of pharmacy counseling on compliance and effectiveness of combination lipid-lowering therapy in patients undergoing coronary artery revascularization: a randomized, controlled trial. Pharmacotherapy. 2000;20: 410-416.

S93. Solomon DK, Portner TS, Bass GEet al. Clinical and economic outcomes in the hypertension and COPD arms of a multicenter outcomes study. J Am Pharm Assoc (Wash). 1998;38: 574-585.

S94. Lowe CJ, Raynor DK, Courtney EA, Purvis J, Teale C. Effects of self medication programme on knowledge of drugs and compliance with treatment in elderly patients. BMJ. 1995;310: 1229-1231.

S95. Lipton HL, Bird JA. The impact of clinical pharmacists' consultations on geriatric patients' compliance and medical care use: a randomized controlled trial. Gerontologist. 1994;34: 307-315.

S96. Lourens H, Woodward MC. Impact of a Medication Card on Compliance in Older People. Australian Journal on Ageing. 1994;13: 72-76.

S97. Tahghighi H, Mortazavi H, Manteghi AA, Armat MR. The effect of comprehensive individual motivational-educational program on medication adherence in elderly patients with bipolar disorders: An experimental study. J Educ Health Promot. 2023;12: 70.

S98. Balli FN, Unsal P, Halil MG, Dogu BB, Cankurtaran M, Demirkan K. Effect of clinical pharmacists' interventions on dementia treatment adherence and caregivers' knowledge. Geriatrics & gerontology international. 2021;21: 506-511.

S99. Gonzalez-Bueno J, Sevilla-Sanchez D, Puigoriol-Juvanteny E, Molist-Brunet N, Codina-Jane C, Espaulella-Panicot J. Improving medication adherence and effective prescribing through a patient-centered prescription model in patients with multimorbidity. Eur J Clin Pharmacol. 2022;78: 127-137.

S100. Vieira LB, Reis AMM, Ramos CA, Reis TMD, Cassiani SHB. The use of an electronic medication organizer device with alarm to improve medication adherence of older adults with hypertension. Einstein (Sao Paulo). 2021;19: eAO6011.

S101. Alkhoshaiban A, Hassan Y, Loganathan M, Alomary M, Morisky DE, Alawwad B. Type II Diabetic Patients' Satisfaction, Medication Adherence, and Glycemic Control after the Application of Pharmacist Counseling Program. Archives of Pharmacy Practice. 2019;10: 127-136.

S102. Lee S, Jiang L, Dowdy D, Hong YA, Ory MG. Effects of the Chronic Disease Self-Management Program on medication adherence among older adults. Transl Behav Med. 2019;9: 380-388.

S103. Doggrell SA. Pilot study, in a rental retirement village, of an "AdherenceCheck" on the management of medicines by the older-aged. International journal of clinical pharmacy. 2017;39: 443-449.

S104. Chen CM, Kuo LN, Cheng KJet al. The effect of medication therapy management service combined with a national PharmaCloud system for polypharmacy patients. Comput Methods Programs Biomed. 2016;134: 109-119.

S105. Mertens A, Brandl C, Miron-Shatz Tet al. A mobile application improves therapy-adherence rates in elderly patients undergoing rehabilitation: A crossover design study comparing documentation via iPad with paper-based control. Medicine (Baltimore). 2016;95: e4446.

S106. Casula M, Tragni E, Piccinelli Ret al. A simple informative intervention in primary care increases statin adherence. Eur J Clin Pharmacol. 2016;72: 227-234.

S107. Hawkins LA, Firek CJ. Testing a novel pictorial medication sheet to improve adherence in veterans with heart failure and cognitive impairment. Heart Lung. 2014;43: 486-493.

S108. Stuurman-Bieze AG, Hiddink EG, van Boven JF, Vegter S. Proactive pharmaceutical care interventions decrease patients' nonadherence to osteoporosis medication. Osteoporos Int. 2014;25: 1807-1812.

S109. Kamimura T, Ishiwata R, Inoue T. Medication reminder device for the elderly patients with mild cognitive impairment. Am J Alzheimers Dis Other Demen. 2012;27: 238-242.

S110. Zhang Y, Lave JR, Donohue JM, Fischer MA, Chernew ME, Newhouse JP. The impact of Medicare Part D on medication adherence among older adults enrolled in Medicare-Advantage products. Med Care. 2010;48: 409-417.

S111. Insel KC, Cole L. Individualizing memory strategies to improve medication adherence. Appl Nurs Res. 2005;18: 199-204.

S112. Raynor DK, Nicolson M, Nunney J, Petty D, Vail A, Davies L. The development and evaluation of an extended adherence support programme by community pharmacists for elderly patients at home. International Journal of Pharmacy Practice. 2000;8: 157-164.

S113. Son KJ, Son HR, Park B, Kim HJ, Kim CB. A Community-Based Intervention for Improving Medication Adherence for Elderly Patients with Hypertension in Korea. Int J Environ Res Public Health. 2019;16.

S114. Brennan TA, Dollear TJ, Hu Met al. An integrated pharmacy-based program improved medication prescription and adherence rates in diabetes patients. Health Aff (Millwood). 2012;31: 120-129.

S115. Bilotta C, Lucini A, Nicolini P, Vergani C. An easy intervention to improve short-term adherence to medications in community-dwelling older outpatients. A pilot non-randomised controlled trial. BMC Health Serv Res. 2011;11: 158.

S116. Smith GE, Lunde AM, Hathaway JC, Vickers KS. Telehealth home monitoring of solitary persons with mild dementia. Am J Alzheimers Dis Other Demen. 2007;22: 20-26.

S117. Kogos SC. Support Groups and Treatment Adherence in a Geriatric Outpatient Clinic. Journal of Clinical Psychology in Medical Settings. 2004;11: 275-282.

S118. Schectman JM, Schorling JB, Nadkarni MM, Voss JD. Can prescription refill feedback to physicians improve patient adherence? Am J Med Sci. 2004;327: 19-24.

S119. Finley PR, Rens HR, Pont JTet al. Impact of a collaborative pharmacy practice model on the treatment of depression in primary care. Am J Health Syst Pharm. 2002;59: 1518-1526.

S120. Wolfe SC, Schirm V. Medication counseling for the elderly: effects on knowledge and compliance after hospital discharge. Geriatr Nurs. 1992;13: 134-138.

S121. Leirer VO, Morrow DG, Tanke ED, Pariante GM. Elders' nonadherence: its assessment and medication reminding by voice mail. Gerontologist. 1991;31: 514-520.

S122. Smith-Ray R, Feng L, Singh Tet al. Pharmacists as clinical care partners: How a pharmacist-led intervention is associated with improved medication adherence in older adults with common chronic conditions. J Manag Care Spec Pharm. 2024;30: 345-351.

S123. Liu J, Shi X, Guo B. Influence of Information-Based Continuous Care on Disease Control and Treatment Compliance of Elderly Diabetic Patients. Evid Based Complement Alternat Med. 2022;2022: 4023123.

S124. Guerard B, Omachonu V, Perez B, Sen B. The Effectiveness of a Comprehensive Wellness Assessment on Medication Adherence in a Medicare Advantage Plan Diabetic Population. J Healthc Manag. 2018;63: 132-141.

S125. Desteghe L, Kluts K, Vijgen Jet al. The Health Buddies App as a Novel Tool to Improve Adherence and Knowledge in Atrial Fibrillation Patients: A Pilot Study. JMIR Mhealth Uhealth. 2017;5: e98.

S126. Lester CA, Mott DA, Chui MA. The Influence of a Community Pharmacy Automatic Prescription Refill Program on Medicare Part D Adherence Metrics. J Manag Care Spec Pharm. 2016;22: 801-807.

S127. Bisharat B, Hafi L, Baron-Epel O, Armaly Z, Bowirrat A. Pharmacist counseling to cardiac patients in Israel prior to discharge from hospital contribute to increasing patient's medication adherence closing gaps and improving outcomes. J Transl Med. 2012;10: 34.

S128. Foreman KF, Stockl KM, Le LBet al. Impact of a text messaging pilot program on patient medication adherence. Clin Ther. 2012;34: 1084-1091.
